# Supplementary material for: Murine models of IDH-wild-type glioblastoma exhibit spatial segregation of tumor initiation and manifestation during evolution
Source: Nat Commun. 2020 Jul 22;11:3669. doi: 10.1038/s41467-020-17382-3 (PMC7376246; doi:10.1038/s41467-020-17382-3)
Supplement: Supplementary file 1 — Supplementary Information [file 41467_2020_17382_MOESM1_ESM.pdf]

Supplementary Information

Murine models of *IDH*-wild-type glioblastoma exhibit spatial segregation of tumor initiation and manifestation during evolution

Li et al.

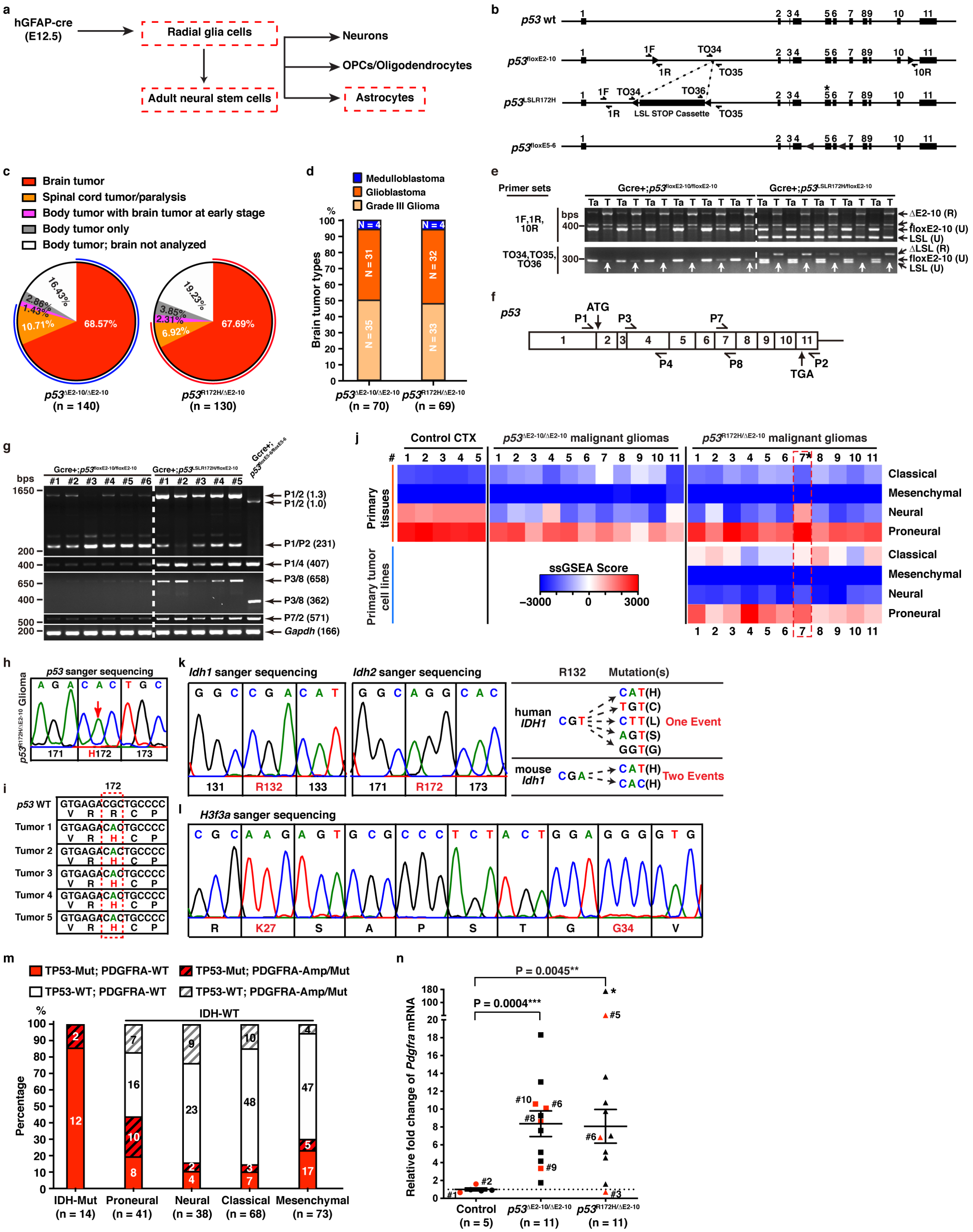

a
hGFAP-cre
(E12.5)
Radial glia cells
Adult neural stem cells
Neurons
OPCs/Oligodendrocytes
Astrocytes
b
p53 wt
p53floxE2-10
p53LSLR172H
p53floxE5-6
1F
1R
TO34
TO35
TO36
10R
LSL STOP Cassette
c
Brain tumor
Spinal cord tumor/paralysis
Body tumor with brain tumor at early stage
Body tumor only
Body tumor; brain not analyzed
68.57%
10.71%
1.43%
2.86%
16.43%
67.69%
6.92%
2.31%
3.85%
19.23%
p53ΔE2-10/ΔE2-10 (n = 140)
p53R172H/ΔE2-10 (n = 130)
d
Medulloblastoma
Glioblastoma
Grade III Glioma
Brain tumor types %
N = 4
N = 31
N = 35
N = 32
N = 33
p53ΔE2-10/ΔE2-10 (n = 70)
p53R172H/ΔE2-10 (n = 69)
e
Primer sets
Gcre+;p53floxE2-10/floxE2-10
Gcre+;p53LSLR172H/floxE2-10
1F,1R, 10R
TO34,TO35, TO36
ΔE2-10 (R)
floxE2-10 (U)
LSL (U)
ΔLSL (R)
f
p53
ATG
TGA
P1 P2 P3 P4 P7 P8
g
Gcre+;p53floxE2-10/floxE2-10
Gcre+;p53LSLR172H/floxE2-10
Gcre+;p53floxE5-6/floxE5-6
P1/2 (1.3)
P1/2 (1.0)
P1/P2 (231)
P1/4 (407)
P3/8 (658)
P3/8 (362)
P7/2 (571)
Gapdh (166)
j
Control CTX
p53ΔE2-10/ΔE2-10 malignant gliomas
p53R172H/ΔE2-10 malignant gliomas
Primary tissues
Primary tumor cell lines
Classical
Mesenchymal
Neural
Proneural
ssGSEA Score
−3000 0 3000
h
p53 sanger sequencing
p53R172H/ΔE2-10 Glioma
171 H172 173
i
p53 WT
Tumor 1
Tumor 2
Tumor 3
Tumor 4
Tumor 5
GTGAGACGCTGCCCC
GTGAGACACTGCCCC
V R R C P
V R H C P
k
Idh1 sanger sequencing
131 R132 133
Idh2 sanger sequencing
171 R172 173
R132
Mutation(s)
human IDH1
CGT
CAT(H)
TGT(C)
CTT(L)
AGT(S)
GGT(G)
One Event
mouse Idh1
CGA
CAT(H)
CAC(H)
Two Events
l
H3f3a sanger sequencing
R K27 S A P S T G G34 V
m
TP53-Mut; PDGFRA-WT
TP53-Mut; PDGFRA-Amp/Mut
TP53-WT; PDGFRA-WT
TP53-WT; PDGFRA-Amp/Mut
IDH-WT
Percentage
IDH-Mut (n = 14)
Proneural (n = 41)
Neural (n = 38)
Classical (n = 68)
Mesenchymal (n = 73)
n
P = 0.0045**
P = 0.0004***
Relative fold change of Pdgfra mRNA
Control (n = 5)
p53ΔE2-10/ΔE2-10 (n = 11)
p53R172H/ΔE2-10 (n = 11)

Supplementary Figure 1. Characterization of *p53*^{R172H}CKO and *p53*^{null}CKO malignant glioma/GBM models.

(a) The lineage relationship between neural stem cells and differentiated cells is illustrated. The hGFAP-cre transgene is expressed in radial glia during embryonic development at embryonic day 12.5 (E12.5), and in type B neural stem cells as well as mature astrocytes in the postnatal brain (all three hGFAP-cre-expressing cell types are marked by dashed boxes). Consequently, the conditional *p53* mutations were targeted into diverse brain cell populations, including those that do not express hGFAP-cre, including all the progeny of neural stem cells, oligodendrocyte precursor cells (OPCs)/oligodendrocytes, and neurons. Targeted cells with long-term self-renewal potentials (e.g. adult neural stem cells) are most susceptible to tumor formation, as accumulation of additional oncogenic driver mutations is required for *p53*-mutant cells to undergo malignant transformation in the brain.

(b) Genetic configuration of three *p53* conditional mutant alleles is illustrated and compared to *p53* wild-type allele. Positions for the genotyping primers are illustrated. * indicates the location of *p53*^{R172H} (exon 5).

(c) The percentage of mice that were analyzed due to brain tumors (red), spinal cord tumor/paralysis (orange), or soft-tissue sarcomas (white, pink and gray) in *p53*^{null}CKO and *p53*^{R172H}CKO mice. Brains from a cohort of mice sacrificed due to soft-tissue sarcomas in the trunk were examined for the presence (pink) or absence (gray) of early-stage brain tumors.

(d) The brain tumor spectrum of *p53*^{null}CKO and *p53*^{R172H}CKO mice is shown.

(e) Genomic DNA isolated from matched tails (Ta) and brain tumors (T, white arrows) of *p53*^{null}CKO and *p53*^{R172H}CKO mice was subjected to PCR analysis for the *p53*$^{floxE2\text{-}10}$ (upper panel) and *p53*^{LSLR172H} (lower panel) alleles. Resultant DNA was labeled as: floxE2-10 (U), non-recombined *p53*$^{floxE2\text{-}10}$ allele; LSL (U), non-recombined *p53*^{LSLR172H} allele; ΔE2-10 (R), recombined *p53*$^{floxE2\text{-}10}$ allele; ΔLSL (R), recombined *p53*^{LSLR172H} allele; and (*), *p53* pseudo band. Tails mainly contained un-recombined alleles. Tumors were predominantly comprised of recombined alleles. Of note, given the relative positions on the *p53* gene, PCR analysis designed for the non-recombined and recombined *p53*$^{floxE2\text{-}10}$ alleles detects the same band for the *p53*^{LSLR172H} allele regardless of recombination (right lanes in the upper panel). However, PCR analysis designed for the non-recombined and recombined the *p53*^{LSLR172H} alleles only detects the non-recombined, but not the recombined, *p53*$^{floxE2\text{-}10}$ alleles (left lanes in the lower panel).

(f) RT-PCR strategy to detect the exons of *p53* gene. Positions for the primers (P1–P4 and P7-P8) are illustrated.

(g) RT-PCR analysis confirms that the correct transcripts are generated from brain tumors in both models. Tumors from both models have the *p53* transcript lacking exons from 2 to 10 generated from the mutant *p53*$^{floxE2\text{-}10}$ allele, while *p53*^{R172H}CKO tumors retain one full-length *p53* transcript. A *p53*$^{\Delta E5\text{-}6}$CKO brain tumor was used as a control.

(h, i) Targeted Sanger sequencing on the entire coding regions of *p53* was performed on *p53*^{R172H}CKO gliomas (n = 5). A representative image shows that the *p53*^{R172H}CKO glioma retained the R172H mutation (**h**). Aside from G515A, the mutation corresponding to R172H, no other mutation sites were identified on *p53* gene in any of these 5 tumors (**i**).

(j) The gene expression subtypes of primary tumor tissues of *p53*^{null}CKO mice (n = 11), and paired tumor and primary tumor-derived cell lines of *p53*^{R172H}CKO mice (n = 11) were predicated with an enrichment-based method called single sample gene set enrichment analysis (ssGSEA). Normal cortex (CTX, n = 5) was used as a control. High score (red) indicates strong similarity, while low score (blue) indicates dissimilarity. Tumor content was confirmed with histological analysis after dissection. Primary tumor #7 (red box, *) was documented by histology with normal tissue contamination after dissection.

(k) Targeted Sanger sequencing on the entire coding regions of *Idh1* and *Idh2* were performed on *p53*^{null}CKO gliomas (n = 10) and *p53*^{R172H}CKO gliomas (*n* = 6). No mutations on *Idh1* and *Idh2* were identified in these tumors. A representative image shows that no mutation was identified at *Idh1* at R132 and *Idh2* at R172 in these tumors of, respectively. Of note, to generate the same mutation, such as R132H, a single event is required for human *IDH1*, while two events are required for mouse *Idh1*.

(l) Targeted Sanger sequencing on the entire coding regions of *H3f3a* was performed on *p53*^{null}CKO gliomas (n = 10) and *p53*^{R172H}CKO gliomas (*n* = 11). No mutation on *H3f3a* was identified in these tumors. A representative image showed that no mutation on *H3f3a* at K27 or G34 sites was identified in this tumor. H3F3A K27M and G34R/V are commonly mutated in pediatric high-grade glioma/GBMs.

(m) The frequency of *TP53* mutations and *PDGFRA* amplification/mutations in *IDH*-mutant and *IDH*-wild type (*IDH*-WT) GBMs from the TCGA datasets [1] were analyzed.

(n) The expression levels of *Pdgfrα* in the primary tumor tissues of *p53*^{null}CKO (n = 11), *p53*^{R172H}CKO (n = 11), and normal cortical tissues (n = 5) from microarray data were compared. Data are presented as dot-plot with Mean ± SEM. Unpaired, two-tailed Student's t-test was used to analyze the statistical. Sample marked with * is a statistical outlier. A group of samples was subjected to both microarray (**n**, red points) and western blotting (Fig. 1**d**) analysis. Source data underlying (**e** and **g**)are provided as a Source data file.

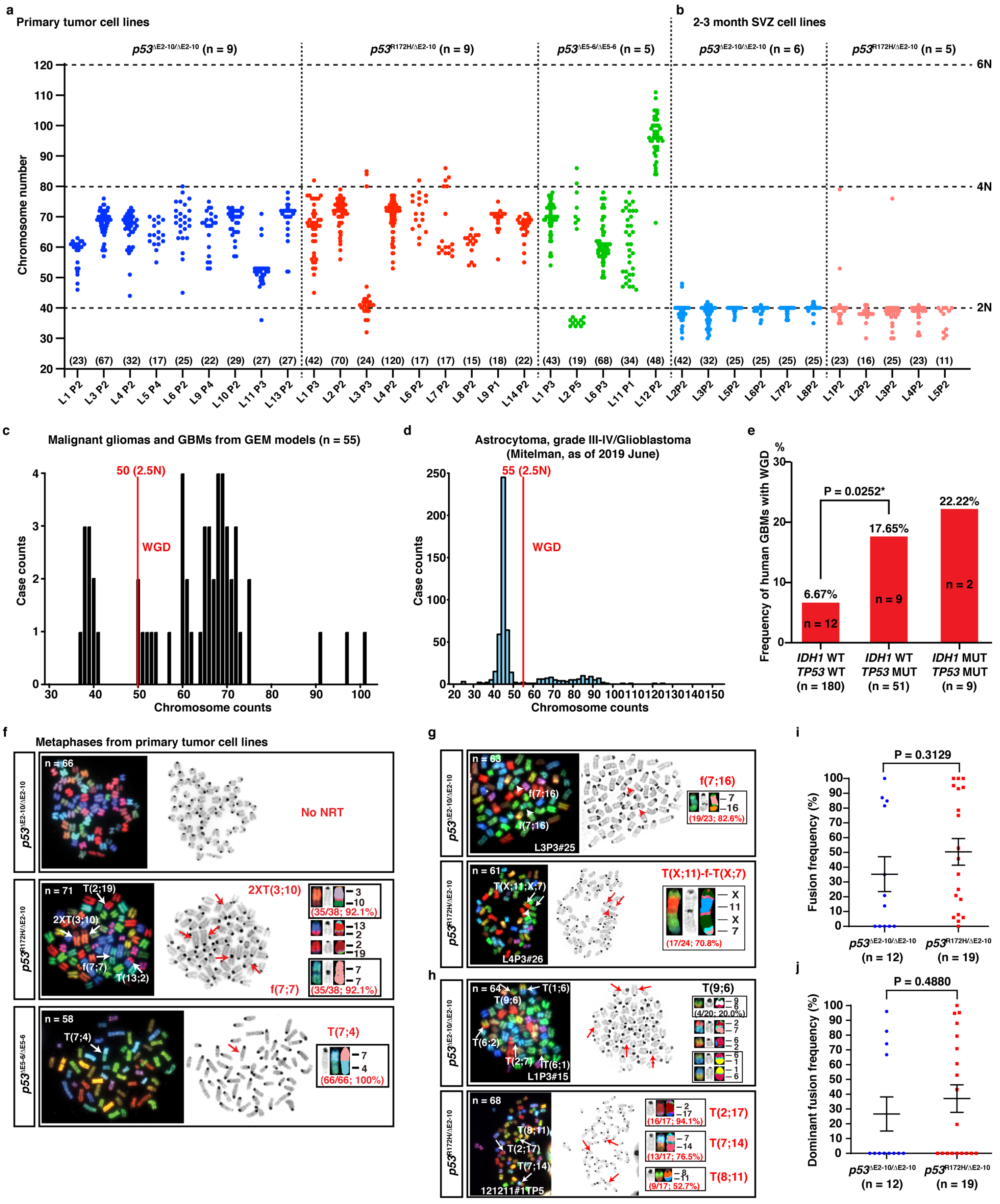

a
Primary tumor cell lines
b
2-3 month SVZ cell lines
p53ΔE2-10/ΔE2-10 (n = 9)
p53R172H/ΔE2-10 (n = 9)
p53ΔE5-6/ΔE5-6 (n = 5)
p53ΔE2-10/ΔE2-10 (n = 6)
p53R172H/ΔE2-10 (n = 5)
Chromosome number
6N
4N
2N
(23) (67) (32) (17) (25) (22) (29) (27) (27) (42) (70) (24) (120) (17) (17) (15) (18) (22) (43) (19) (68) (34) (48) (42) (32) (25) (25) (25) (25) (23) (16) (25) (23) (11)
L1 P2, L3 P2, L4 P2, L5 P4, L6 P2, L9 P4, L10 P2, L11 P3, L13 P2, L1 P3, L2 P2, L3 P3, L4 P2, L6 P2, L7 P2, L8 P2, L9 P1, L14 P2, L1 P3, L2 P5, L6 P3, L11 P1, L12 P2, L2P2, L3P2, L5P2, L6P2, L7P2, L8P2, L1P2, L2P2, L3P2, L4P2, L5P2
c
Malignant gliomas and GBMs from GEM models (n = 55)
50 (2.5N)
WGD
Case counts
Chromosome counts
d
Astrocytoma, grade III-IV/Glioblastoma
(Mitelman, as of 2019 June)
55 (2.5N)
WGD
Case counts
Chromosome counts
e
%
Frequency of human GBMs with WGD
P = 0.0252*
6.67%
17.65%
22.22%
n = 12
n = 9
n = 2
IDH1 WT TP53 WT (n = 180)
IDH1 WT TP53 MUT (n = 51)
IDH1 MUT TP53 MUT (n = 9)
f
Metaphases from primary tumor cell lines
p53ΔE2-10/ΔE2-10
n = 66
No NRT
p53R172H/ΔE2-10
n = 71
T(2;19)
2XT(3;10)
f(7;7)
T(13;2)
(35/38; 92.1%)
p53ΔE5-6/ΔE5-6
n = 58
T(7;4)
(66/66; 100%)
g
p53ΔE2-10/ΔE2-10
n = 63
f(7;16)
L3P3#25
(19/23; 82.6%)
p53R172H/ΔE2-10
n = 61
T(X;11;X;7)
T(X;11)-f-T(X;7)
L4P3#26
(17/24; 70.8%)
h
p53ΔE2-10/ΔE2-10
n = 64
T(1;6)
T(9;6)
T(6;2)
T(2;7)
T(6;1)
L1P3#15
(4/20; 20.0%)
p53R172H/ΔE2-10
n = 68
T(8;11)
T(2;17)
T(7;14)
121211#1TP5
(16/17; 94.1%)
(13/17; 76.5%)
(9/17; 52.7%)
i
P = 0.3129
Fusion frequency (%)
p53ΔE2-10/ΔE2-10 (n = 12)
p53R172H/ΔE2-10 (n = 19)
j
P = 0.4880
Dominant fusion frequency (%)
p53ΔE2-10/ΔE2-10 (n = 12)
p53R172H/ΔE2-10 (n = 19)

Supplementary Figure 2. Whole-genome duplication (WGD) and acquisition of clonal non-reciprocal translocations (cNRTs) were observed in malignant gliomas/GBMs from *p53*^{R172H}CKO and *p53*$^{ΔE5-6}$CKO models.

(a, b) Chromosomal profiles were compared between primary tumor-derived cell lines (**a**) and primary 2-3-month-old SVZ-derived cell lines (**b**) from three *p53*CKO models using karyotyping assay as indicated. Numbers in parentheses indicate the number of metaphases analyzed for each cell line. Each dot represents a metaphase. All cell lines were analyzed prior to 5 passages (<P5).

(c) Histogram showing distribution of chromosome counts of malignant gliomas from p53-mutant driven GEM models used in this study. The red line indicates a chromosome count of 50 which is about ploidy 2.5 of normal mouse chromosomes.

(d) Histogram showing distribution of chromosome counts of malignant gliomas including Grade III Astrocytoma and Glioblastoma (IV) from the Mitelman database (data downloaded as of 7/2019). The red line indicates a chromosome count of 55 which is about ploidy 2.5 of normal human chromosomes excluding sex chromosomes.

(e) The proportion of human GBMs exhibiting WGD were compared between *IDH*-wild type GBMs - *TP53*-wild type and *TP53*-mutant - and *IDH1*-mutant GBMs. Fisher's exact test was used to analyze the statistical difference.

(f) Representative SKY images show NRTs (arrows) in *p53*^{R172H}CKO and *p53*$^{ΔE5-6}$CKO gliomas but not in *p53*^{null}CKO gliomas. The frequency of each dominant NRT was shown.

(g) Clonal fusions (head-to-head; head-to-tail or tail-to-tail) (arrow heads) were identified in gliomas from both *p53*^{null}CKO model (n = 4, out of 12) and *p53*^{R172H}CKO model (n = 8, out of 19). Notably, one *p53*^{R172H}CKO glioma shows both cNRT (arrows) and fusion (arrowhead) in the same abnormal chromosomal structure (lower panel).

(h) The metaphases from a *p53*^{null}CKO glioma with the most severe chromosomal structural abnormality is shown (upper panel). None of the 5 NRTs (arrows) identified in this cell line was clonal. In contrast, 3 different cNRTs were identified in a *p53*^{R172H}CKO glioma (lower panel).

(i, j) The percentage of cells from each tumor sample carrying a fusion (**i**) and the percentage of cells carrying the dominant fusion (the fusion with the highest frequency in a tumor sample) (**j**) were compared between *p53*^{null}CKO (n = 12) and *p53*^{R172H}CKO (n = 19) tumors. Each dot represents a tumor sample. Data are presented as dot-plot with Mean ± SEM. Unpaired, two-tailed Student's t-test was used for statistical analysis.

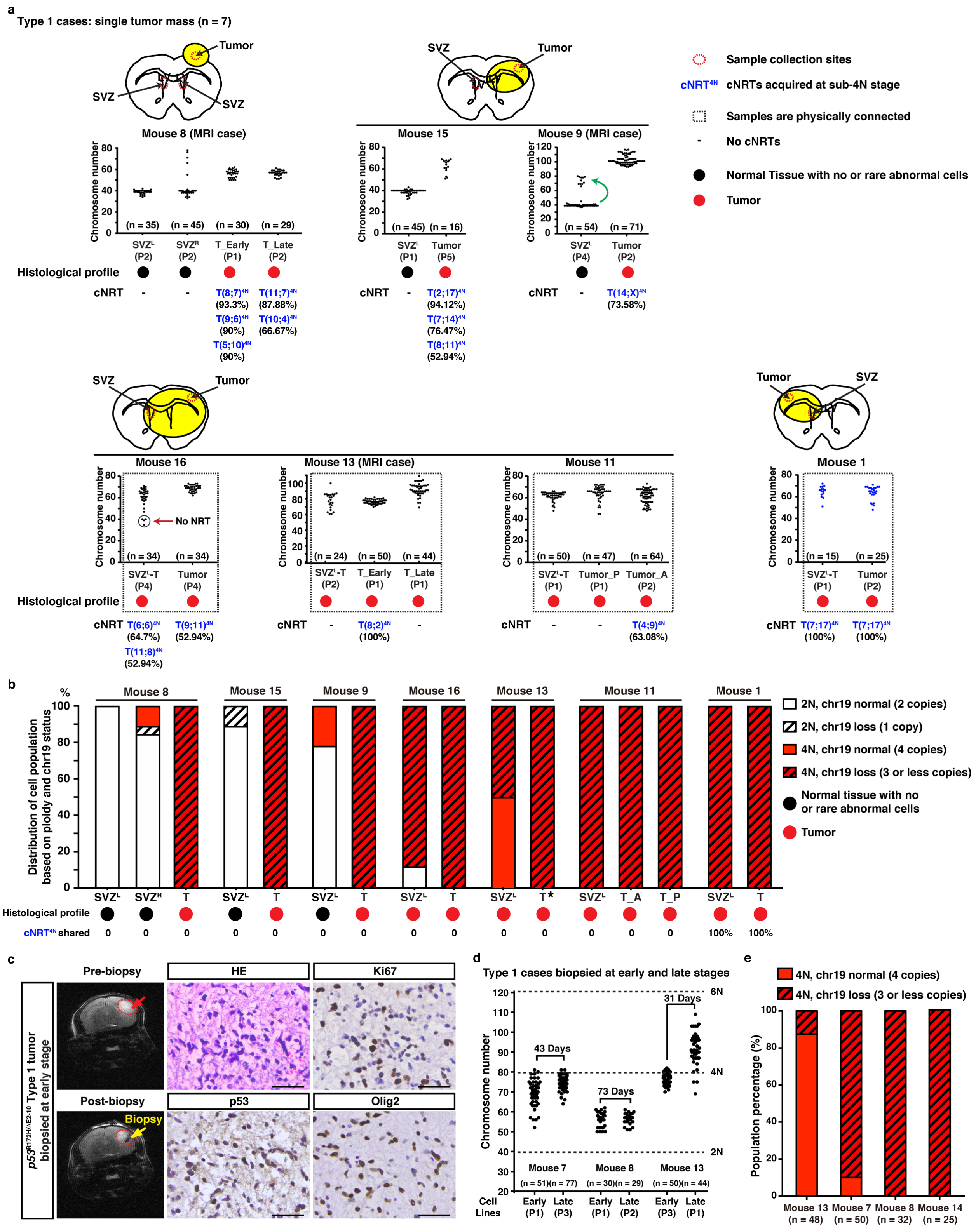
a
Type 1 cases: single tumor mass (n = 7)
Tumor
SVZ
SVZ
Mouse 8 (MRI case)
Mouse 15
Mouse 9 (MRI case)
Sample collection sites
cNRT4N cNRTs acquired at sub-4N stage
Samples are physically connected
No cNRTs
Normal Tissue with no or rare abnormal cells
Tumor
Chromosome number
SVZL (P2) SVZR (P2) T_Early (P1) T_Late (P2)
SVZL (P1) Tumor (P5)
SVZL (P4) Tumor (P2)
Histological profile
cNRT
T(8;7)4N (93.3%) T(9;6)4N (90%) T(5;10)4N (90%)
T(11;7)4N (87.88%) T(10;4)4N (66.67%)
T(2;17)4N (94.12%) T(7;14)4N (76.47%) T(8;11)4N (52.94%)
T(14;X)4N (73.58%)
Mouse 16
Mouse 13 (MRI case)
Mouse 11
Mouse 1
No NRT
SVZL-T (P4) Tumor (P4)
SVZL-T (P2) T_Early (P1) T_Late (P1)
SVZL-T (P1) Tumor_P (P1) Tumor_A (P2)
SVZL-T (P1) Tumor (P2)
T(6;6)4N (64.7%) T(11;8)4N (52.94%) T(9;11)4N (52.94%)
T(8;2)4N (100%)
T(4;9)4N (63.08%)
T(7;17)4N (100%) T(7;17)4N (100%)
b
Mouse 8 Mouse 15 Mouse 9 Mouse 16 Mouse 13 Mouse 11 Mouse 1
Distribution of cell population based on ploidy and chr19 status
2N, chr19 normal (2 copies)
2N, chr19 loss (1 copy)
4N, chr19 normal (4 copies)
4N, chr19 loss (3 or less copies)
Normal tissue with no or rare abnormal cells
Tumor
SVZL SVZR T SVZL T SVZL T SVZL T SVZL T* SVZL T_A T_P SVZL T
cNRT4N shared 0 0 0 0 0 0 0 0 0 0 0 0 0 100% 100%
c
Pre-biopsy
Post-biopsy
HE
Ki67
p53
Olig2
Biopsy
p53R172H/ΔE2-10 Type 1 tumor biopsied at early stage
d
Type 1 cases biopsied at early and late stages
43 Days
73 Days
31 Days
6N 4N 2N
Mouse 7 Mouse 8 Mouse 13
Cell Lines
Early (P1) Late (P3) Early (P1) Late (P2) Early (P3) Late (P1)
e
4N, chr19 normal (4 copies)
4N, chr19 loss (3 or less copies)
Population percentage (%)
Mouse 13 (n = 48) Mouse 7 (n = 50) Mouse 8 (n = 32) Mouse 14 (n = 25)

Supplementary Figure 3. One-phase tumor evolution in *p53*^{R172H}CKO Type 1 cases.

(a) Cartoons show the sampling strategy in Type 1 cases (n = 7) with single-mass tumors in *p53*^{R172H}CKO mice. Biopsy samples were analyzed by histopathological analysis (Ki67 and Olig2 labeling, except Mouse 1), and histological profile of each sample was determined as tumor (red dot under the sample name) or relatively normal (black dot under the sample name). SKY analysis was performed on early passages of paired primary tumor and SVZ cell lines from each case. Chromosome number and clonal NRT (cNRT) information for each sample are illustrated. The passage(s) and number of metaphases analyzed for each sample are indicated. Each dot represents a metaphase. Frequency of each cNRT is shown under the name of each sample. "–", samples without cNRT. Notably, all Type 1 cases contained one or more cNRT(s) acquired at the 4N stage, termed as cNRT^{4N} (blue). Each of the cNRT^{4N}s identified in six Type 1 cases (Mouse 8, 15, 9, 16, 13 and 11) were not shared between different segments of the same tumors or between different cases. An arrow (green) in SVZ sample of Mouse 9 indicates NRTs sharing was observed in a small population with near-2N and sub-4N genome, but this NRT was not shared with other tumor samples in that mouse. In one Type 1 case (Mouse 1), the cNRT^{4N} was shared in all the metaphases in both samples (blue dots).

(b) Ploidy and chr19 status at single cell level are illustrated by SKY analysis of early-passage cells derived from matched parenchymal tumors and SVZ tissues in *p53*^{R172H}CKO Type 1 cases (n = 7). Cartoons (below) show the histology of parenchymal tumor versus SVZ with sampling strategy (red dotted circles). The frequency of cells shared with the same cNRT^{4N} from different sites of each of the 7 Type 1 cases is shown. *: the tumor cells in Mouse 13 contained sub-6N genome.

(c) Adjacent sections from one representative *p53*^{R172H}CKO Type 1 tumor brain biopsied at tumor early stage (mouse without symptoms) were subjected to histopathological analysis (H&E staining and Ki67, p53 and Olig2 labeling). Scale bars, 100 µm.

(d) Chromosome number comparison between tumors biopsied at early and late stages is illustrated in 3 Type 1 cases. The age difference between the two biopsies (early and late) is indicated.

(e) Ploidy and chr19 status at single cell level are illustrated for the tumors biopsied at early stages in 4 Type 1 cases.

Type 2 cases: multiple lesions were initiated at spatially distinct brain regions, may with or without tumor merge at end-stages

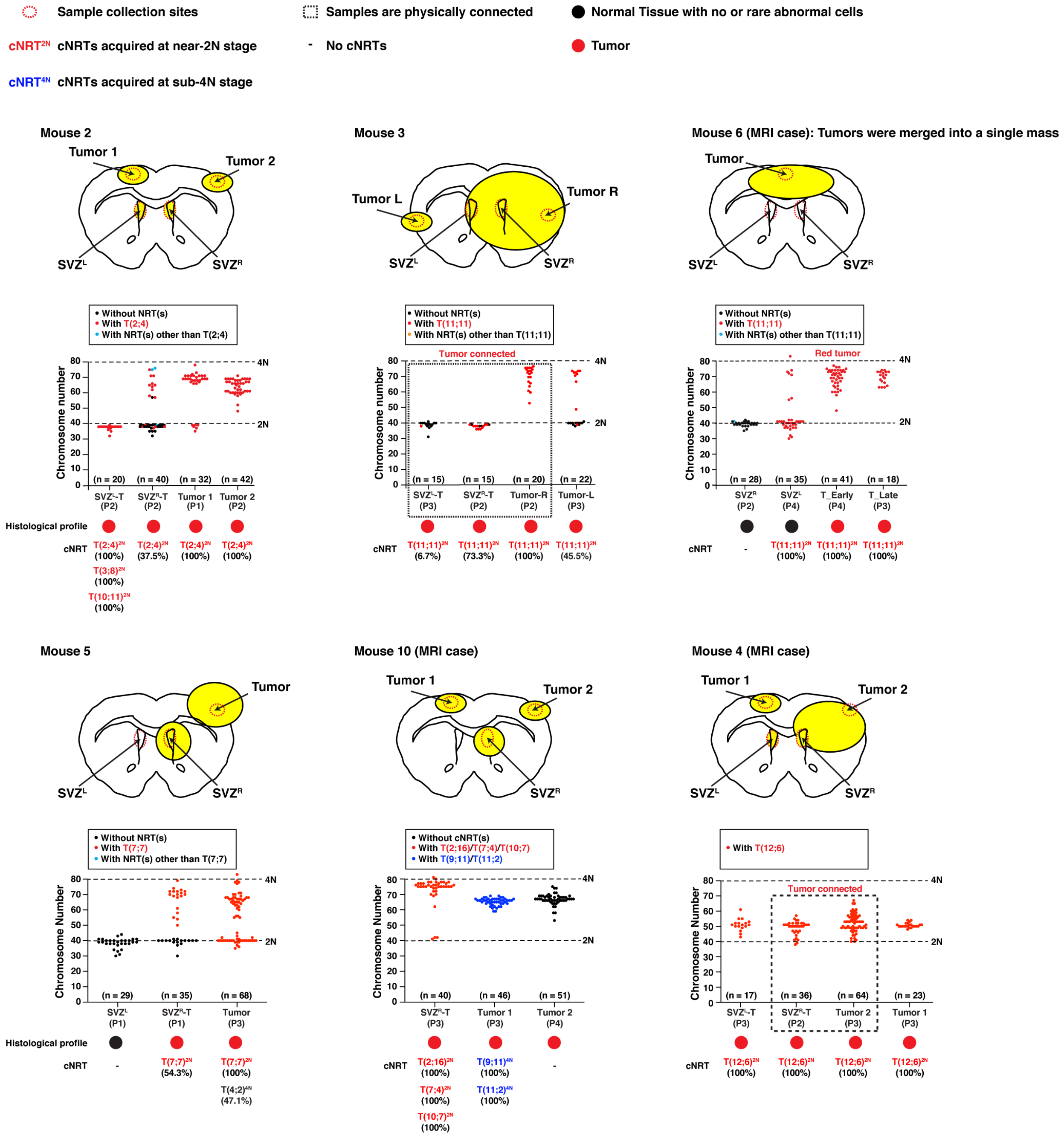

Supplementary Figure 4. Chromosomal abnormalities in SVZ- and parenchymal tumor-derived samples of Type 2 cases were identified by SKY analysis.

Cartoons show the sampling strategy in Type 2 cases (n = 6) with tumor masses occurred at multiple spatially segregated sites in *p53*^{R172H}CKO mice. Biopsy samples were analyzed by histopathological analysis (Ki67 and Olig2 labeling), and histological profile of each sample was determined as tumor (red dot under the sample name) or relatively normal (black dot under the sample name). SKY analysis was performed on early passages of paired primary tumor and SVZ cell lines from each case. Chromosome number and cNRT information for each sample are illustrated. The passage(s) and number of metaphases analyzed for each sample are indicated. Each dot represents a metaphase. Frequency of each cNRT is shown under the name of each sample. "–", samples without cNRT. Notably, all Type 2 cases contained cNRTs acquired at 2N stage, termed as cNRT^{2N} (red dots). The metaphases with shared unique cNRT^{2N} in each of the 5 cases were colored with red. The only exception is Mouse 10, which contained both cNRT^{2N} (SVZ tumor, red dots) and cNRT^{4N} (parenchyma Tumor 1, blue dots).

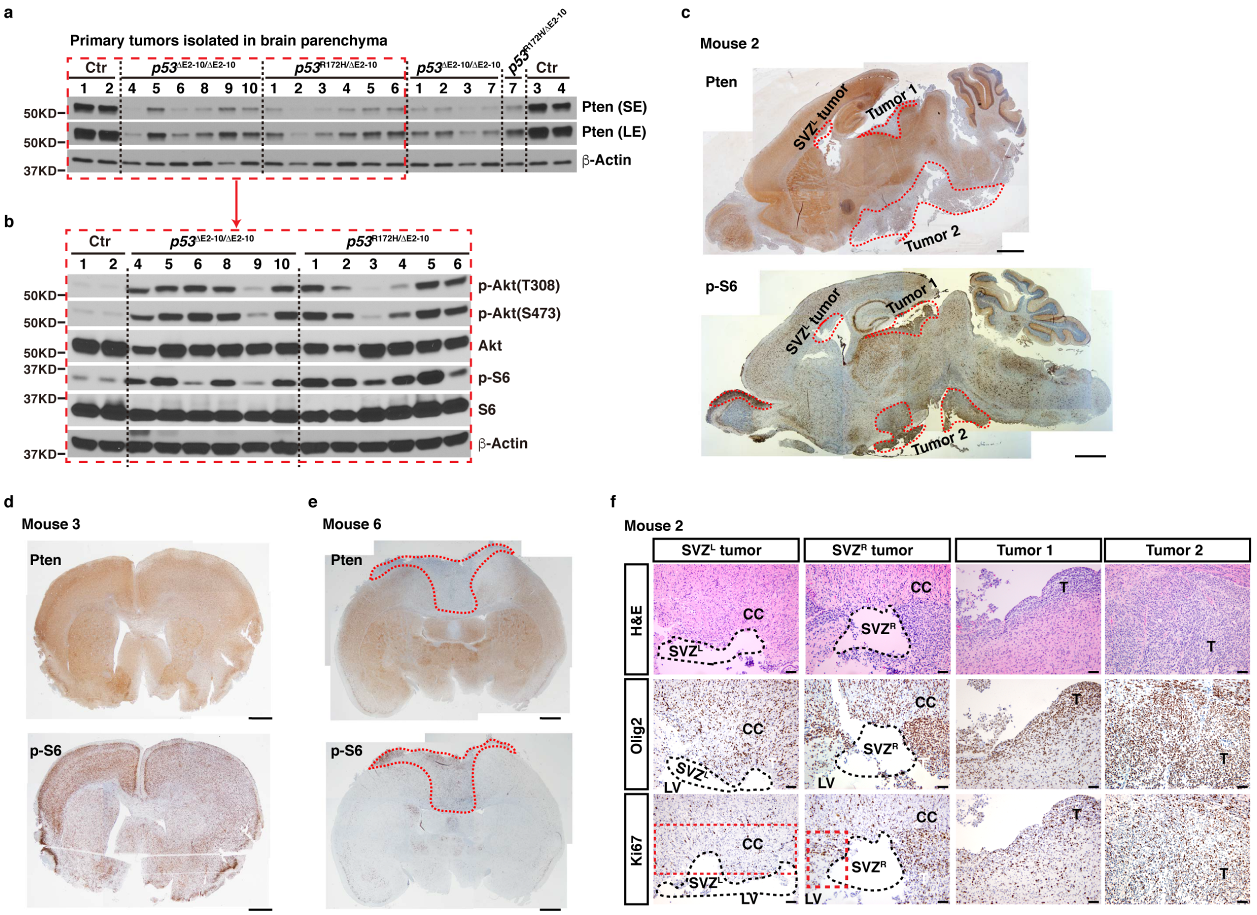

Supplementary Figure 5. Loss of *Pten* and activation of PI3K/Akt/mTORC1 signaling in malignant gliomas/GBMs from the brain parenchyma of *p53*CKO mice.

(a) Pten protein level was determined in malignant gliomas/GBMs samples isolated from brain parenchyma in *p53*^{null}CKO (n = 10) and *p53*^{R172H}CKO (n = 7) mice by Western blotting analysis. Normal adult cortical tissues from hGFAP-cre+; *p53*$^{floxE2\text{-}10/+}$ (n = 4) mice were used as controls (Ctr).

(b) A set of samples from (a), labelled with red box, was subjected to Western blotting analysis for p-Akt and p-S6 protein levels. Note that the β-actin image is the same as shown in Fig. 1d, which is from the same experiment.

(c-e) Representative images show Pten and p-S6 staining in three Type 2 cases - Mouse 2 (**c**), Mouse 3 (**d**) and Mouse 6 (**e**) from *p53*^{R172H}CKO model. Loss of Pten, accompanied by activation of phospho-S6, an indicator of PI3K/Akt/mTORC1 signaling, was observed in tumors in the brain parenchyma. Conversely, SVZ tumors or normal brain regions in Mouse 2 and Mouse 6 exhibited high Pten expression, accompanied by low p-S6 expression. Of note, certain *Pten* wild-type brain

areas, including neurons in the hippocampus and cerebellum in (**c**), exhibited high levels of p-S6 expression. This was in contrast to malignant gliomas in the corresponding SVZ and brain parenchyma, thus suggesting Pten-independent regulation of PI3K/Akt/mTORC1 signaling pathway in neurons, but not in GBMs. Additionally, tumors in Mouse 3 exhibited robust p-S6 expression, despite having normal Pten expression (likely due to the *Pik3ca*^{H1047R} mutation).

(f) Representative images show H&E, Olig2 and Ki67 staining of 4 spatially segregated tumor regions from Mouse 2 brain. Dashed lines mark tissue dissection sites. Lower proliferation areas in both SVZ regions are highlighted by a red box.

LV, Lateral ventricle; CC, Corpus Callosum; T, Tumor; SE, short exposure; LE, long exposure. Scale bars, 100 µm (**f**) and 1 mm (**c**-**e**). Source data underlying (**a** and **b**) are provided as a Source data file.

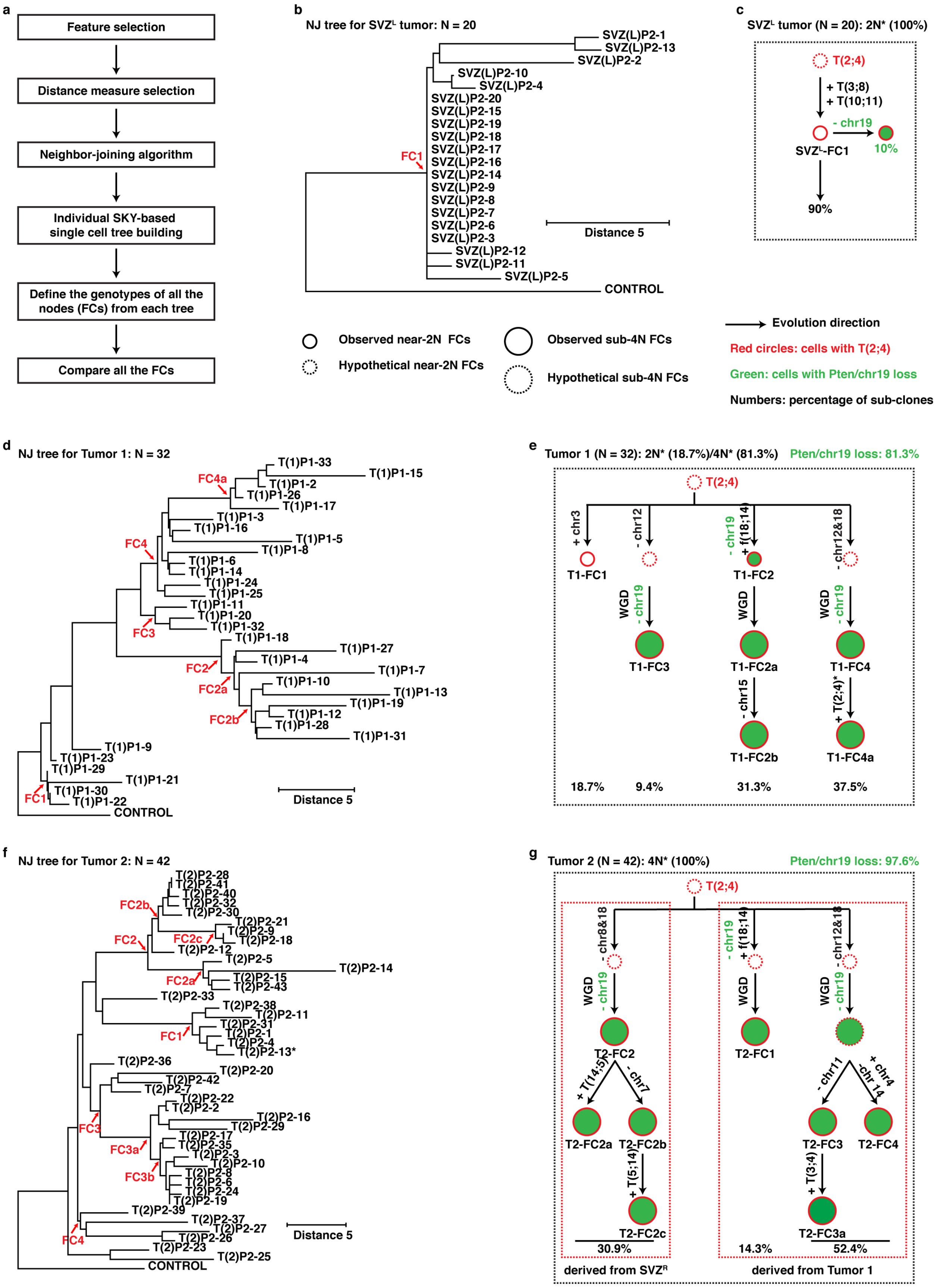
a
Feature selection
Distance measure selection
Neighbor-joining algorithm
Individual SKY-based single cell tree building
Define the genotypes of all the nodes (FCs) from each tree
Compare all the FCs
b
NJ tree for SVZ^L tumor: N = 20
SVZ(L)P2-1
SVZ(L)P2-13
SVZ(L)P2-2
SVZ(L)P2-10
SVZ(L)P2-4
SVZ(L)P2-20
SVZ(L)P2-15
SVZ(L)P2-19
SVZ(L)P2-18
SVZ(L)P2-17
SVZ(L)P2-16
SVZ(L)P2-14
SVZ(L)P2-9
SVZ(L)P2-8
SVZ(L)P2-7
SVZ(L)P2-6
SVZ(L)P2-3
SVZ(L)P2-12
SVZ(L)P2-11
SVZ(L)P2-5
CONTROL
FC1
Distance 5
c
SVZ^L tumor (N = 20): 2N* (100%)
T(2;4)
+ T(3;8)
+ T(10;11)
- chr19
SVZ^L-FC1
10%
90%
Observed near-2N FCs
Hypothetical near-2N FCs
Observed sub-4N FCs
Hypothetical sub-4N FCs
Evolution direction
Red circles: cells with T(2;4)
Green: cells with Pten/chr19 loss
Numbers: percentage of sub-clones
d
NJ tree for Tumor 1: N = 32
T(1)P1-33
T(1)P1-15
T(1)P1-2
T(1)P1-26
T(1)P1-17
T(1)P1-3
T(1)P1-16
T(1)P1-5
T(1)P1-8
T(1)P1-6
T(1)P1-14
T(1)P1-24
T(1)P1-25
T(1)P1-11
T(1)P1-20
T(1)P1-32
T(1)P1-18
T(1)P1-27
T(1)P1-4
T(1)P1-7
T(1)P1-10
T(1)P1-13
T(1)P1-19
T(1)P1-12
T(1)P1-28
T(1)P1-31
T(1)P1-9
T(1)P1-23
T(1)P1-29
T(1)P1-21
T(1)P1-30
T(1)P1-22
CONTROL
FC4a
FC4
FC3
FC2
FC2a
FC2b
FC1
Distance 5
e
Tumor 1 (N = 32): 2N* (18.7%)/4N* (81.3%)
Pten/chr19 loss: 81.3%
T(2;4)
+ chr3
- chr12
- chr19
+ t(18;14)
- chr12&18
T1-FC1
T1-FC2
WGD
- chr19
T1-FC3
T1-FC2a
T1-FC4
- chr15
+ T(2;4)*
T1-FC2b
T1-FC4a
18.7%
9.4%
31.3%
37.5%
f
NJ tree for Tumor 2: N = 42
T(2)P2-28
T(2)P2-41
T(2)P2-40
T(2)P2-32
T(2)P2-30
T(2)P2-21
T(2)P2-9
T(2)P2-18
T(2)P2-12
T(2)P2-5
T(2)P2-14
T(2)P2-15
T(2)P2-43
T(2)P2-33
T(2)P2-38
T(2)P2-11
T(2)P2-31
T(2)P2-1
T(2)P2-4
T(2)P2-13*
T(2)P2-36
T(2)P2-20
T(2)P2-42
T(2)P2-7
T(2)P2-22
T(2)P2-2
T(2)P2-16
T(2)P2-29
T(2)P2-17
T(2)P2-35
T(2)P2-3
T(2)P2-10
T(2)P2-8
T(2)P2-6
T(2)P2-24
T(2)P2-19
T(2)P2-39
T(2)P2-37
T(2)P2-27
T(2)P2-26
T(2)P2-23
T(2)P2-25
CONTROL
FC2b
FC2
FC2c
FC2a
FC1
FC3
FC3a
FC3b
FC4
Distance 5
g
Tumor 2 (N = 42): 4N* (100%)
Pten/chr19 loss: 97.6%
T(2;4)
- chr8&18
- chr19
+ t(18;14)
- chr12&18
WGD
- chr19
T2-FC2
T2-FC1
+ T(14;5)
- chr7
- chr11
+ chr4
-chr 14
T2-FC2a
T2-FC2b
T2-FC3
T2-FC4
+ T(5;14)
+ T(3;4)
T2-FC2c
T2-FC3a
30.9%
14.3%
52.4%
derived from SVZ^R
derived from Tumor 1

Supplementary Figure 6. SKY-based single-cell phylogenetic trees in Mouse 2.

(a) Schematic illustration of the steps for building SKY-based single-cell/clone phylogenetic trees and clonal evolution interpretations.

(b, d and f) SKY-based single-cell/clone phylogenetic tree was generated with all metaphases in SVZ^L tumor (**b**), Tumor 1 (**d**) and Tumor 2 (**f**) in Mouse 2. It was generated by using the neighbor-joining algorithm with customized feature and distance measure selection. The positions of major clusters are indicated by arrows.

(c, e and g) The genotype of each founder cell/clones (FCs) on the phylogenetic trees was identified and their clonal relationship was illustrated in SVZ^L tumor (**c**), Tumor 1 (**e**) and Tumor 2 (**g**). Major chromosomal events of the FCs in each branch are shown. Note that the 7 FCs in Tumor 1 could be the result of three clonally related progeny of the CA (SVZ^R-FC1), after acquiring additional chromosomal events: chr3 gain (which generates T1-FC1); chromosomal fusion [f(18;14)] (which generates the progeny of T1-FC2) and chr12 loss (which generates the progeny of T1-FC3 and T1-FC4), respectively. For Tumor 2, the 9 FCs could be traced back to three clonally related progeny of the CA, SVZR-FC1. While the FC (T2-FC2) directly came from the predicted SVZ^R-FC2 (with additional chr12 loss), the other two (T2-FC1 and the progeny of T2-FC3/4) most likely came from Tumor 1 (T1-FC2a and T1-FC4) by acquiring additional genetic events.

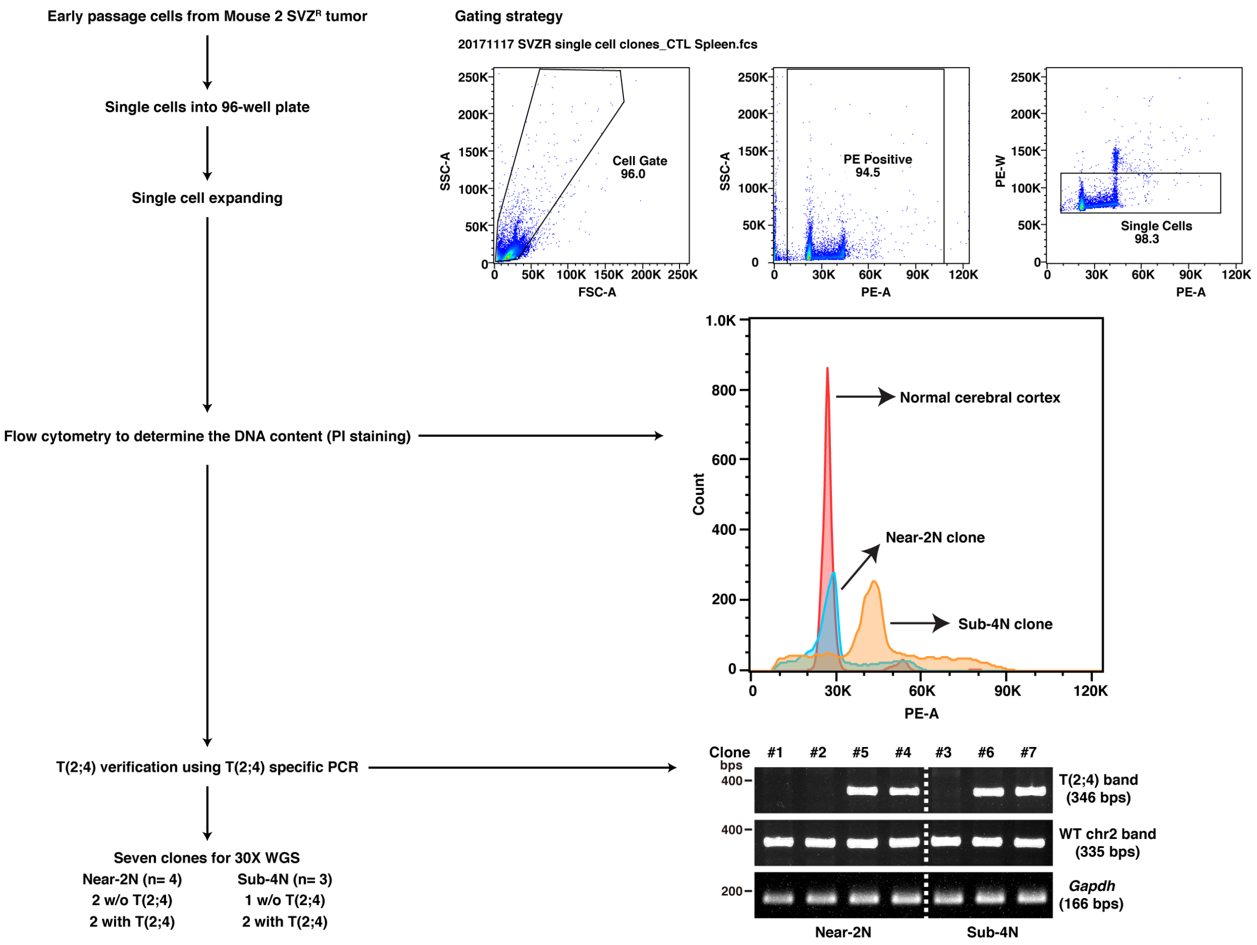

Supplementary Figure 7. Flowchart of the experimental procedures for the WGS analysis of single-cell-derived tumor samples from SVZ^{R} tumor in Mouse 2.

Briefly, single cells were individually seeded into 96-well plates and expanded. Flow cytometry was used to determine the ploidy based on DNA content. Gating strategy is shown on top (right), representative image shows a clone with near-2N genome and a clone with sub-4N genome compared with normal cerebral cortex cells with diploid genome (middle right). Then the clones were subjected for T(2;4) identification by PCR analysis. The PCR results are shown in the bottom (right). Lastly, several clones with the defined ploidy and T(2;4) status were subjected to whole-genome sequencing (30X). Source data are provided as a Source data file.

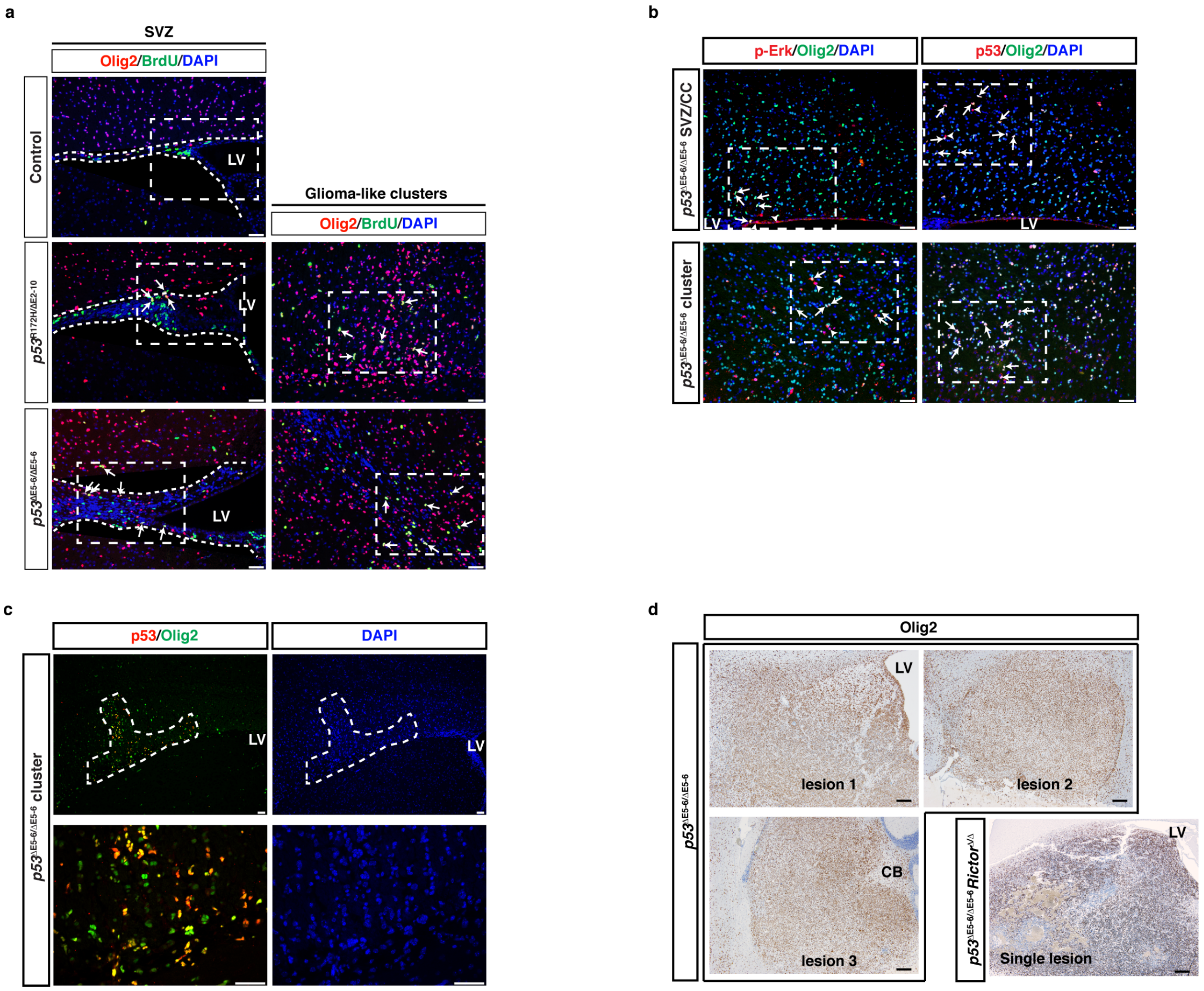

Supplementary Figure 8. RAS/MAPK and PI3K/AKT/mTORC pathways are associated with local expansion in the SVZ and distant dispersal from the SVZ, respectively.

(a-c) Control and mutant mice at age 5-7 months were injected with BrdU to detect proliferating cells in tumor-free brains. White dashed lines in (**a**) marks the areas of SVZ and/or RMS in the stem-cell niche.

(a) Representative images show co-labelling of Olig2 and BrdU in the SVZ and early glioma-like clusters in *p53*^{R172H}CKO and *p53*$^{\Delta E5\text{-}6}$CKO brains, but not in control brains. Arrows indicate the abnormal Olig2^{+}BrdU^{+} cells. High magnification of boxed areas are shown in Fig. 7f.

(b) Representative images show co-labelling of p-Erk and Olig2 in the SVZ (**b**, top left) and early glioma-like clusters (**b**, bottom left), as well as co-labelling of p53$^{\Delta E5\text{-}6}$ and Olig2 (**b**, right) in

p53$^{\Delta E5\text{-}6}$CKO brains. Arrows indicate p-Erk^{+}Olig2^{+} or p53$^{\Delta E5\text{-}6}$Olig2^{+} cells. Arrowheads indicate p-Erk^{+}Olig2^{-} cells. High magnification of boxed areas are shown in Fig. 7 g and h.

(c) Representative images showed a single early glioma-like cluster located outside of the SVZ niche in a *p53*$^{\Delta E5\text{-}6}$CKO brain. High magnification images of abnormal p53$^{\Delta E5\text{-}6}$Olig2^{+} cells were shown in the bottom panel. White dashed circle marks the edge of the cluster.

(d) Representative images show Olig2 staining in a brain with multifocal lesions from the *p53*$^{\Delta E5\text{-}6}$CKO model versus a brain with a single lesion from the *p53*$^{\Delta E5\text{-}6}$*Rictor*$^{\Delta/\Delta}$CKO model. Olig2 expression is a glioma specific marker, confirming lesion 3 as a glioma, not a medulloblastoma, despite in the cerebellar localization.

LV, Lateral ventricle; CB, cerebellum. Scale bars: 50 µm (**a-c**); 25 µm (**d**).
