# Supplementary material for: Murine models of IDH-wild-type glioblastoma exhibit spatial segregation of tumor initiation and manifestation during evolution
Source: Nat Commun. 2020 Jul 22;11:3669. doi: 10.1038/s41467-020-17382-3 (PMC7376246; doi:10.1038/s41467-020-17382-3)
Supplement: Supplementary file 3 — Description of Additional Supplementary Files [file 41467_2020_17382_MOESM3_ESM.docx]

File Name: Supplementary Movie 1

Description: 3D MRI reconstruction shows the temporal tumor growth in a Type 1 case from *p53*^R172H^CKO model.

File Name: Supplementary Movie 2

Description: 3D MRI reconstruction shows the temporal tumor growth in a Type 2 case from *p53*^R172H^CKO model.

File Name: Supplementary Movie 3

Description: 3D MRI reconstruction shows the temporal tumor growth in a Type 2 case from *p53*^∆E5-6^CKO model.

File Name: Supplementary Movie 4

Description: 3D MRI reconstruction shows the temporal tumor growth in a Type 2 case from *p53*^R172H^CKO models.
