# Supplementary material for: Murine models of IDH-wild-type glioblastoma exhibit spatial segregation of tumor initiation and manifestation during evolution
Source: Nat Commun. 2020 Jul 22;11:3669. doi: 10.1038/s41467-020-17382-3 (PMC7376246; doi:10.1038/s41467-020-17382-3)
Supplement: Supplementary file 8 — Reporting Summary [file 41467_2020_17382_MOESM8_ESM.pdf]

nature research

Corresponding author(s): YUAN ZHU

Last updated by author(s): Jun 9, 2020

Reporting Summary

Nature Research wishes to improve the reproducibility of the work that we publish. This form provides structure for consistency and transparency in reporting. For further information on Nature Research policies, see our Editorial Policies and the Editorial Policy Checklist.

Statistics

For all statistical analyses, confirm that the following items are present in the figure legend, table legend, main text, or Methods section.

n/a	Confirmed	
☐	☒	The exact sample size (*n*) for each experimental group/condition, given as a discrete number and unit of measurement
☐	☒	A statement on whether measurements were taken from distinct samples or whether the same sample was measured repeatedly
☐	☒	The statistical test(s) used AND whether they are one- or two-sided<br>*Only common tests should be described solely by name; describe more complex techniques in the Methods section.*
☐	☒	A description of all covariates tested
☐	☒	A description of any assumptions or corrections, such as tests of normality and adjustment for multiple comparisons
☐	☒	A full description of the statistical parameters including central tendency (e.g. means) or other basic estimates (e.g. regression coefficient) AND variation (e.g. standard deviation) or associated estimates of uncertainty (e.g. confidence intervals)
☐	☒	For null hypothesis testing, the test statistic (e.g. F, t, r) with confidence intervals, effect sizes, degrees of freedom and P value noted<br>*Give P values as exact values whenever suitable.*
☐	☒	For Bayesian analysis, information on the choice of priors and Markov chain Monte Carlo settings
☐	☒	For hierarchical and complex designs, identification of the appropriate level for tests and full reporting of outcomes
☐	☒	Estimates of effect sizes (e.g. Cohen's d, Pearson's r), indicating how they were calculated

Our web collection on statistics for biologists contains articles on many of the points above.

Software and code

Policy information about availability of computer code

Data collection	All the microscope images for immunostaining were acquired by fluorescence microscope (Olympus BX-63) with the software CellSens Entry (Olympus, version 1.11). SKY images were captured using HiSKYV spectrum imaging systems from ASI (version 7). All the MRI images were acquired on a 9.4T, 16 cm horizontal bore (Agilent Technologies, Inc., Santa Clara, CA) Direct Drive system, Image reconstruction and digital image analysis were done using software algorithms developed in Matlab (The MathWorks, Natick, MA, version 2016b). Flow cytometry data was acquired using the BD FACS Canto II with BD FACSDiva Software (version 6.1.3).
Data analysis	HiSKYV from ASI were used to analyze the SKY data (version 7). We developed R codes and used RStudio (version 1.2.5033), R package “APE” (version 5.2) and MEGAX (version 0.1, for MAC) to generate SKY-based phylogenetic trees. GraphPad 6 and 8 were used for all statistical analysis. MATHLAB and VnmrJ software (version 3.2) were used MRI analysis. Burrows-Wheeler Aligner (BWA, version 0.7.8); SAMtools13 (version 1.0); Picard (version 1.111); GATK14 (version 3.8); ANNOVAR15 (version 2015Dec) and control-FREEC (version 9.9) were used for WGS data analysis. FlowJo (version 10.6.1) was used for DNA content analysis. Adobe photoshop (version CS6) and ImageJ (version 1.52q) was used for image processing. Adobe illustrator (version CS6) was used for figure assembling.

For manuscripts utilizing custom algorithms or software that are central to the research but not yet described in published literature, software must be made available to editors and reviewers. We strongly encourage code deposition in a community repository (e.g. GitHub). See the Nature Research guidelines for submitting code & software for further information.

Data

Policy information about availability of data

All manuscripts must include a data availability statement. This statement should provide the following information, where applicable:

- Accession codes, unique identifiers, or web links for publicly available datasets
- A list of figures that have associated raw data
- A description of any restrictions on data availability

The gene expression data have been deposited in the GEO database under the accession code GSE152071. The whole genome sequencing data have been deposited in the National Center for Biotechnology Information (NCBI) Sequence Read Archive (BioProject accession no. PRJNA638264). The source data underlying Figs 1d; 3b; 4d, e (upper panels); 6f-k; 7b and Supplementary Figs 1e, g; 5a, b; 7 are provided as a Source Data file. All the other data supporting the findings of this study are available within the article and its supplementary information files and from the corresponding author upon reasonable request. A reporting summary for this article is available as a Supplementary Information file.

Field-specific reporting

Please select the one below that is the best fit for your research. If you are not sure, read the appropriate sections before making your selection.

☒ Life sciences ☐ Behavioural & social sciences ☐ Ecological, evolutionary & environmental sciences

For a reference copy of the document with all sections, see nature.com/documents/nr-reporting-summary-flat.pdf

Life sciences study design

All studies must disclose on these points even when the disclosure is negative.

Sample size	Sample sizes were not chosen based on pre-specificed effect size but selected based on commonly adopted standards in the field. Histology, protein analysis and genetic analysis included at least 5 mice per GEM model. Growth curve and MRI analysis used at least 10 animals for the experimental mutants. The sample size for each individual experiment/analysis are shown in the figures, figure legends or Methods section.
Data exclusions	No data were excluded from the analysis.
Replication	All experiments were performed under standard and clearly defined conditions. Results were confirmed by inclusion of numerous independent biological replicates. For chromosome and SKY analysis, 20~80 metaphases per cell line were randomly imaged and analyzed. Each demonstrated immunohistochemical and immunofluorescent image was representative of three or more cases (tumors) of the indicated subtype. Western blots and q-PCR were done for at least three individual experiments (except for q-PCR in figure 4e which has two repeats) and one representative blot/image was shown in the figures or supplementary figures.
Randomization	Histology and genetic analysis were conducted for all mutant mice. Western blots were conducted on a set of randomly selected animals. Animals were selected for MRI, WGS and SKY prior to development of symptoms, and thus growth patterns were random.
Blinding	Blinding was not possible for histology as genotyping was necessary for all experiments. MRI, WGS and SKY were conducted by independent investigators prior to data analysis, with each investigator blind to the other experimental outcomes.

Reporting for specific materials, systems and methods

We require information from authors about some types of materials, experimental systems and methods used in many studies. Here, indicate whether each material, system or method listed is relevant to your study. If you are not sure if a list item applies to your research, read the appropriate section before selecting a response.

Materials & experimental systems

n/a	Involved in the study	
☐	☒	Antibodies
☐	☒	Eukaryotic cell lines
☒	☐	Palaeontology and archaeology
☐	☒	Animals and other organisms
☒	☐	Human research participants
☒	☐	Clinical data
☒	☐	Dual use research of concern

Methods

n/a	Involved in the study	
☒	☐	ChIP-seq
☐	☒	Flow cytometry
☐	☒	MRI-based neuroimaging

Antibodies

Antibodies used	The primary antibodies used in this study for immunohistochemistry and immunofluorescence experiments are: rabbit anti-p53 (NCL-p53-CM5p, 1:1000, Leica Biosystems), mouse anti-Ki67 (550609, 1:500, BD Pharmingen), rabbit anti-Olig2 (AB9610, 1:2000, EMD

Millipore), Guinea pig anti-Olig2 (1:10000, a kind gift of homemade antibody from Dr. B. Novitch), mouse anti-GFAP (556330, 1:2000, BD Pharmingen), rabbit anti-Pten (9559S, 1:1000, Cell Signaling), rabbit anti-p-S6 (5364S, 1:2000, Cell Signaling), rat anti-BrdU (ab6326, 1:500, Abcam), rabbit anti-p-Erk (9101S, 1:2000, Cell Signaling), mouse anti-Ascl1 (556604, 1:100-1:200, BD PharMingen), rabbit anti-Ezh2 (5246S, 1:1,000-1:2,000, Cell Signaling) and rabbit anti-BLBP (ab32423, 1:200, Abcam).
The primary antibodies used for western blotting analysis are: rabbit anti-p53 (NCL-p53-CM5p, 1:1000, Leica Biosystems), rabbit anti-p-Akt(T308) (2965S, 1:1000, Cell Signaling), rabbit anti-p-Akt(S473) (4060L, 1:1000, Cell Signaling), rabbit anti-Akt (9272S, 1:2000, Cell Signaling), rabbit anti-Pten (9559S, 1:1000, Cell Signaling), rabbit anti-p-S6 (5364S, 1:2000, Cell Signaling), rabbit anti-S6 (2217S, 1:2000, Cell Signaling), mouse anti-p120 (Anti-Ras-GAP, 610040, 1:1000, BD Biosciences), rabbit anti-Nf1 (SC-67, 1:1000, Santa Cruz Biotechnology) and mouse anti-β-Actin (A5316, 1:20000, Sigma-Aldrich).

Validation

All antibodies used for western blotting analysis were validated by including appropriate molecular weight markers and determining if the protein band had the expected molecular weight. For antibodies used for immunohistochemistry and immunofluorescence experiments, antibody specificity was tested by adding positive and negative controls and checking their staining patterns according to the data sheet from the company and literatures in the field.

Eukaryotic cell lines

Policy information about cell lines

Cell line source(s)

Primary cell lines were established from tumors and/or SVZs from the GEM models described in this manuscript.

Authentication

The primary cell lines were not further authenticated.

Mycoplasma contamination

Our lab has routine testing to make sure no mycoplasma contamination is detected in the cell culture system. The primary cell lines used in this study were not specifically tested for mycoplasma contamination.

Commonly misidentified lines
(See ICLAC register)

No cell lines used in this study were found in the database of commonly misidentified cell lines that is maintained by ICLAC and NCBI Biosample.

Animals and other organisms

Policy information about studies involving animals; ARRIVE guidelines recommended for reporting animal research

Laboratory animals

The mice used in this study were included in the Methods section and figure legends section. The control and mutant mice of p53nullCKO (p53ΔE2-10CKO, hGFAP-cre+;p53floxE2-10/floxE2-10); p53R172HCKO (hGFAP-cre+;p53LSLR172H/floxE2-10); p53ΔE5-6CKO (hGFAP-cre+;p53floxE5-6/floxE5-6); p53ΔE5-6RictorΔ/+CKO (hGFAP-cre+;p53floxE5-6/floxE5-6;Rictorflox/+) and p53ΔE5-6RictorΔ/ΔCKO (hGFAP-cre+;p53floxE5-6/floxE5-6;Rictorflox/flox) were used. All mice were maintained in the mixed backgrounds of C57Bl6 and 129S1/Svj. Both male and female mice were analyzed either at end-stages of brain tumors/body tumors or at early-stages indicated in the related figure legends. All mice in this study were cared for according to the guidelines approved by the Animal Care and Use Committees of the University of Michigan at Ann Arbor and Children's National Research Institute at Washington DC. Mice were housed according to a 12-h light (6am)/dark (6pm) cycle at 74F, at a constant relative humidity of 40%, with free access to water and rodent standard chow diet prior to the experiments.

Wild animals

No wild animals were used in this study.

Field-collected samples

No field-collected samples were used in this study.

Ethics oversight

The study is compliant with all relevant ethical regulations for animal experiments. All the experimental protocols were approved by the Animal Care and Use Committees of the University of Michigan at Ann Arbor and Children's National Research Institute at Washington DC.

Note that full information on the approval of the study protocol must also be provided in the manuscript.

Flow Cytometry

Plots

Confirm that:

- [x] The axis labels state the marker and fluorochrome used (e.g. CD4-FITC).
- [x] The axis scales are clearly visible. Include numbers along axes only for bottom left plot of group (a 'group' is an analysis of identical markers).
- [x] All plots are contour plots with outliers or pseudocolor plots.
- [x] A numerical value for number of cells or percentage (with statistics) is provided.

Methodology

Sample preparation

Sample preparation information was provided in the Methods section, under the topic of "Cell dispersal, fixation, staining and flow cytometry".

Instrument

BD FACS Canto II

Software	BD FACSDiva Software (version 6.1.3) for data acquiring and FlowJo (Version 9 or 10) for data analysis.
Cell population abundance	PI was used to label DNA content of all fixed cells.
Gating strategy	Only the obvious debris was gating out. Peaks in the PI histogram plot indicates the relative intensity of DNA content. Normal spleen and normal cerebral cortex tissue were used as controls to show the normal diploid DNA content. Then the peaks from all tumor cell lines were compared to the control (normal cerebral cortex tissue) to predicate the ploidy of each tumor cell line.

[x] Tick this box to confirm that a figure exemplifying the gating strategy is provided in the Supplementary Information.

Magnetic resonance imaging

Experimental design

Design type	The experimental procedure was provided in details in Methods section, under topic "MRI scans, image-guided biopsy and image analysis".
Design specifications	MR images were acquired bi-monthly prior to tumor formation, weekly until biopsy and weekly thereafter until the animals were sacrificed or became moribund. The image-guided biopsy was described in the methods part.
Behavioral performance measures	n/a.

Acquisition

Imaging type(s)	T1 and T2-weighted images
Field strength	9.4 Tesla small animal MRI
Sequence & imaging parameters	Supplementary Information/Methods, under section "MRI scans, image-guided biopsy and image analysis".
Area of acquisition	Whole brain scan

Diffusion MRI [ ] Used [x] Not used

Preprocessing

Preprocessing software	Volumes of interest (VOIs) were manually contoured around the hyper-intense portion of the tumors on the T2-weighted images for tumor volume measurements using in-house software (The Center for Molecular Imaging, University of Michigan, Ann Arbor) running in MATLAB (MathWorks, Natick, MA)
Normalization	n/a
Normalization template	n/a
Noise and artifact removal	n/a
Volume censoring	n/a

Statistical modeling & inference

Model type and settings	n/a
Effect(s) tested	n/a

Specify type of analysis: [ ] Whole brain [ ] ROI-based [ ] Both

Statistic type for inference (See Eklund et al. 2016)	n/a
Correction	n/a

Models & analysis

n/a	Involved in the study
[x]	[ ] Functional and/or effective connectivity
[x]	[ ] Graph analysis
[x]	[ ] Multivariate modeling or predictive analysis
