# Supplementary material for: Murine models of IDH-wild-type glioblastoma exhibit spatial segregation of tumor initiation and manifestation during evolution
Source: Nat Commun. 2020 Jul 22;11:3669. doi: 10.1038/s41467-020-17382-3 (PMC7376246; doi:10.1038/s41467-020-17382-3)
Supplement: Supplementary file 9 — Source data [file 41467_2020_17382_MOESM9_ESM.pdf]

Figure 1d

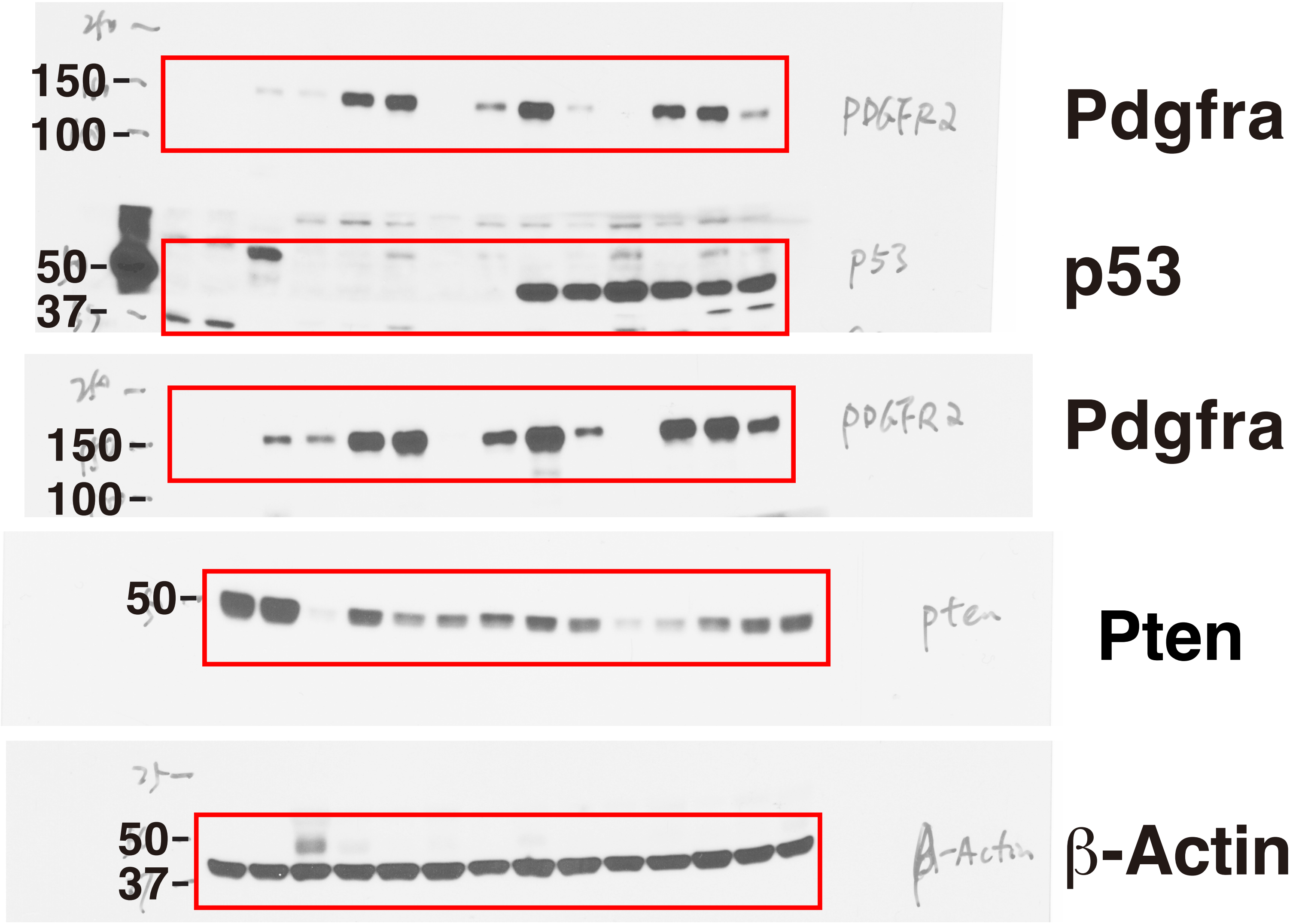

Uncropped immunoblots from Figure 1

Figure 3b

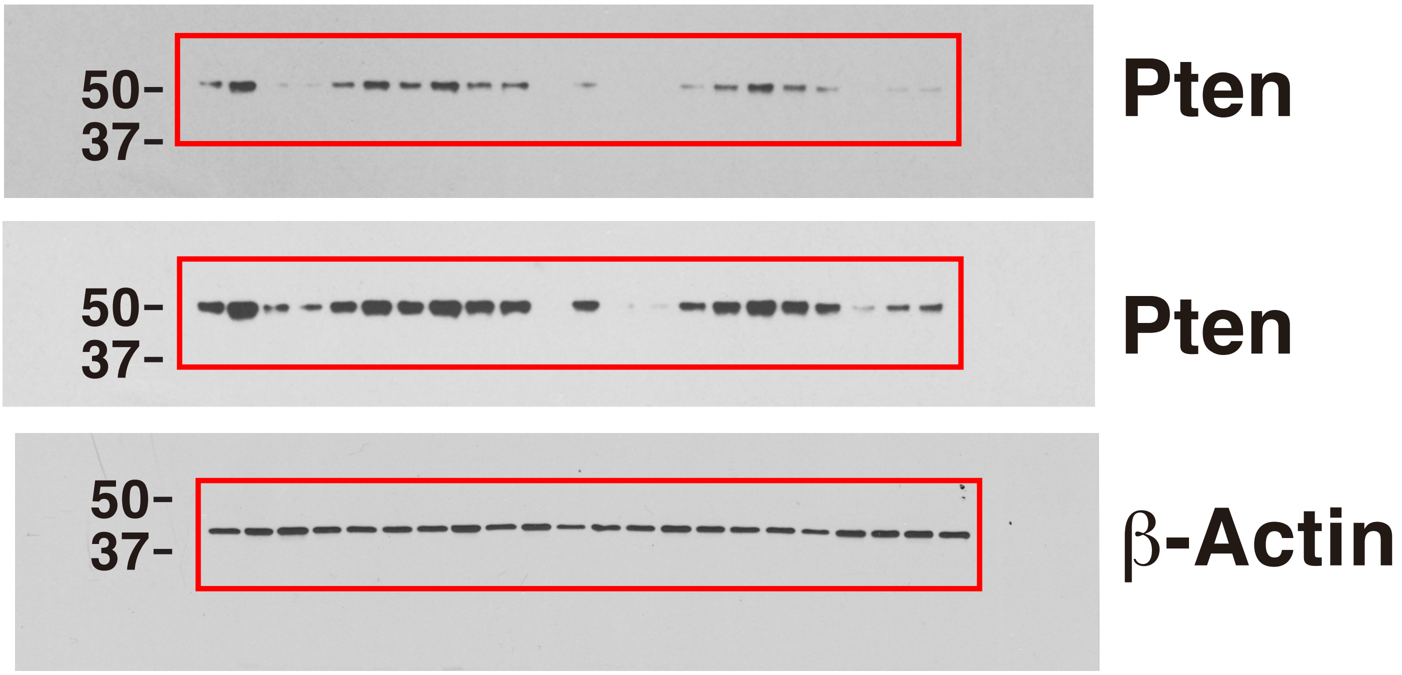

Uncropped immunoblots from Figure 3

Figure 4d

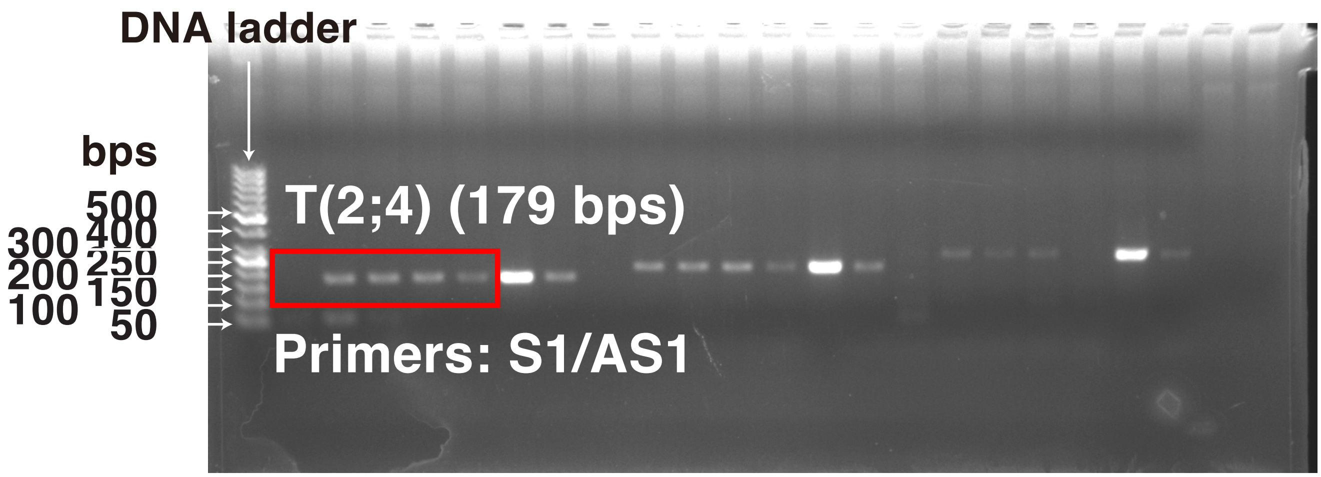

Figure 4e

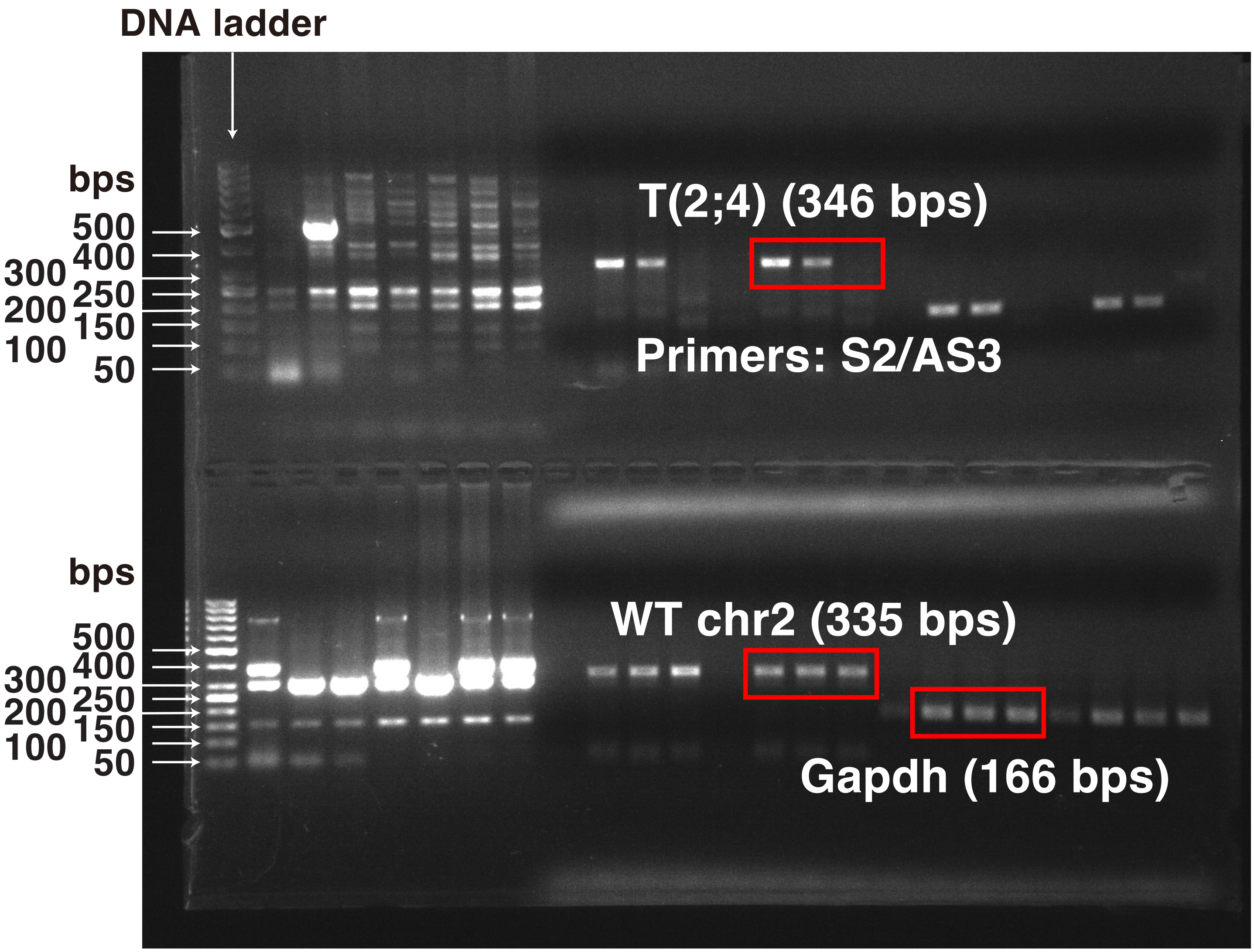

Uncropped DNA gel results from Figure 4

Figure 7b

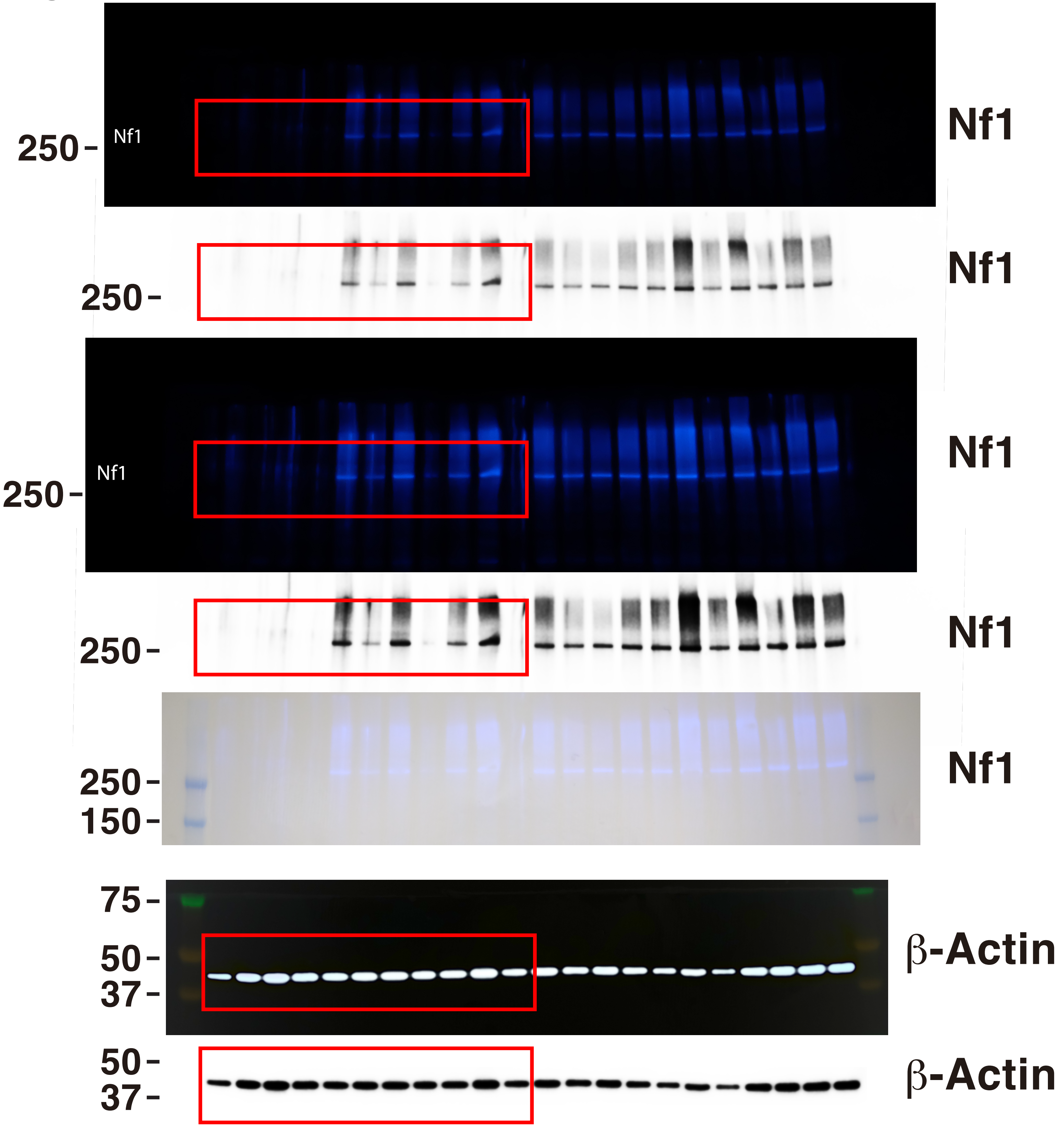

These original images were generated by the imaging machine (KwikQuant™ Imager, Kindle Biosciences, LLC). Then the images were converted to black & white images using the Adobe Photoshop software. During the converting process, we adjusted brightness and contrast of the images.

Uncropped immunoblots from Figure 7

Supplementary Figure 1e

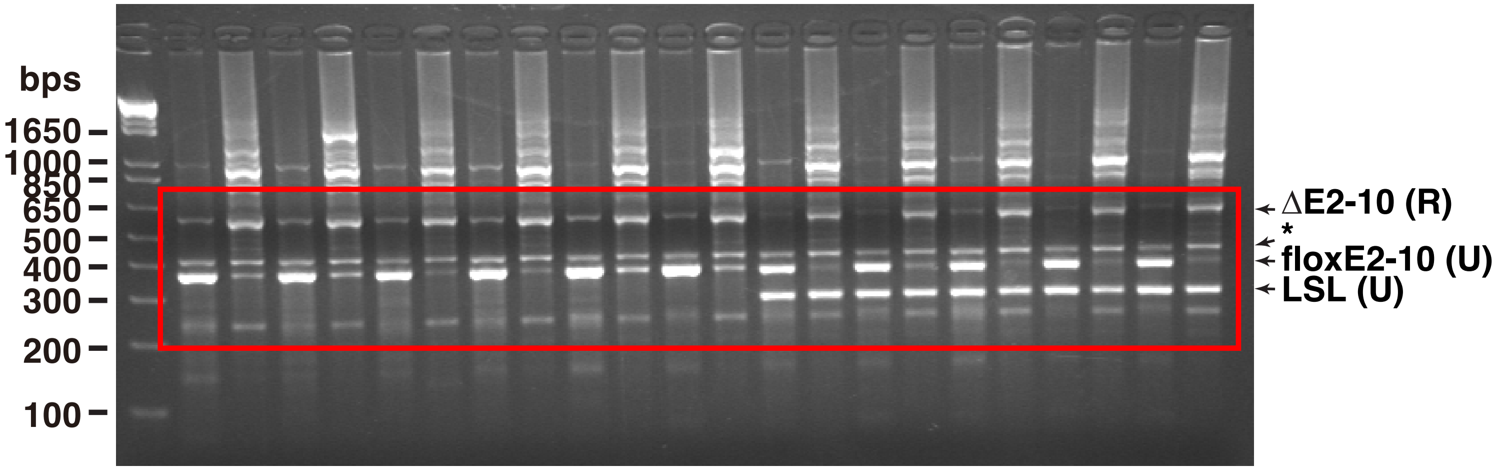

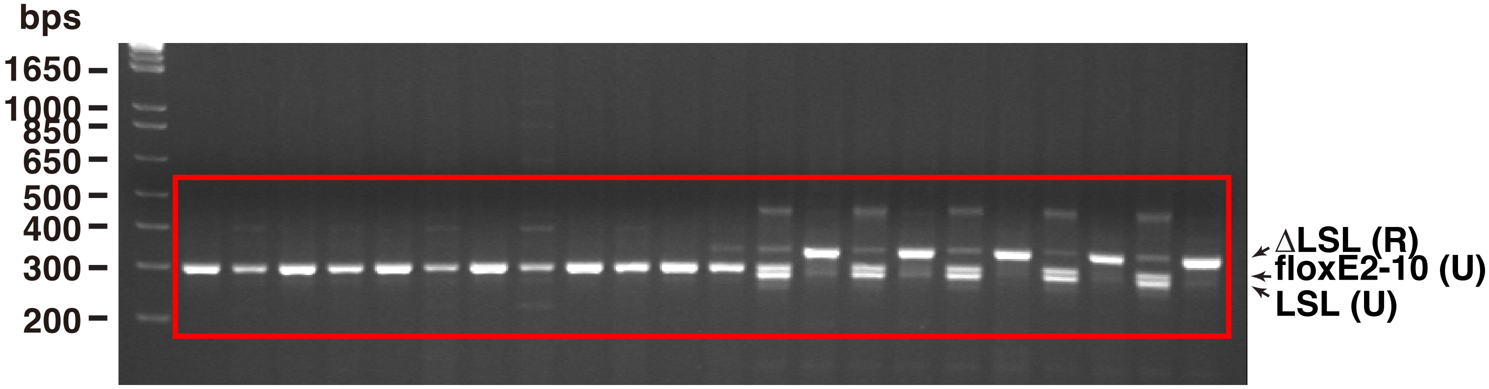

Re-run the samples with lower amount of PCR products

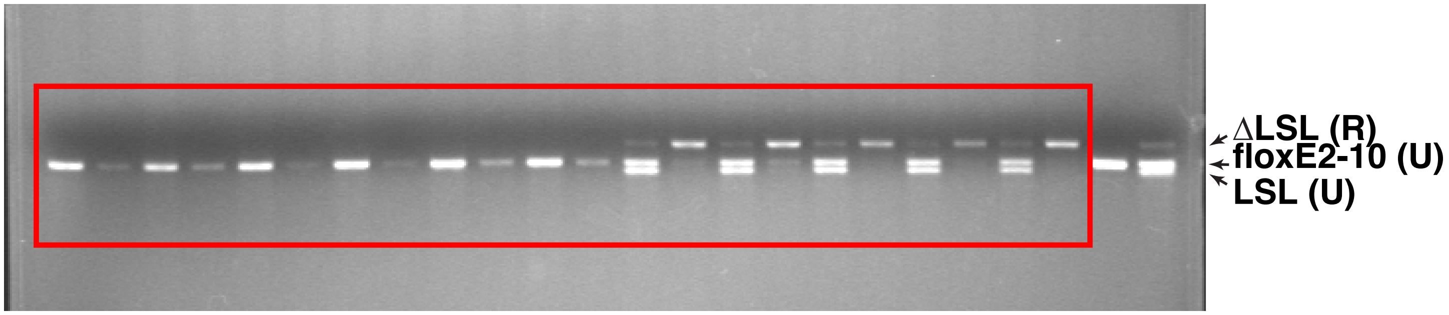

Uncropped DNA gel results from Supplementary Figure 1

Supplementary Figure 1g

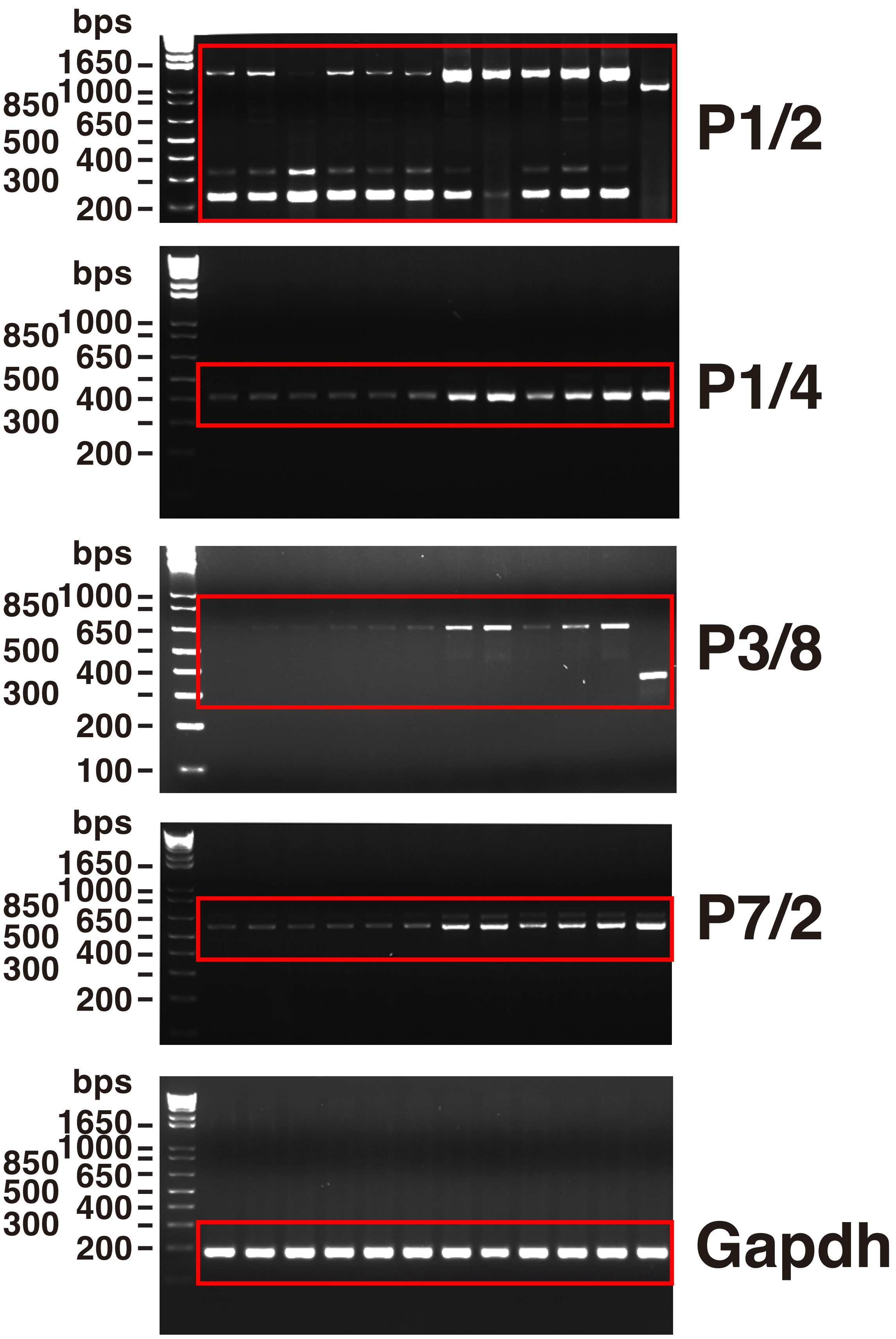

Uncropped DNA gel results from Supplementary Figure 1

Supplementary Figure 5a, b

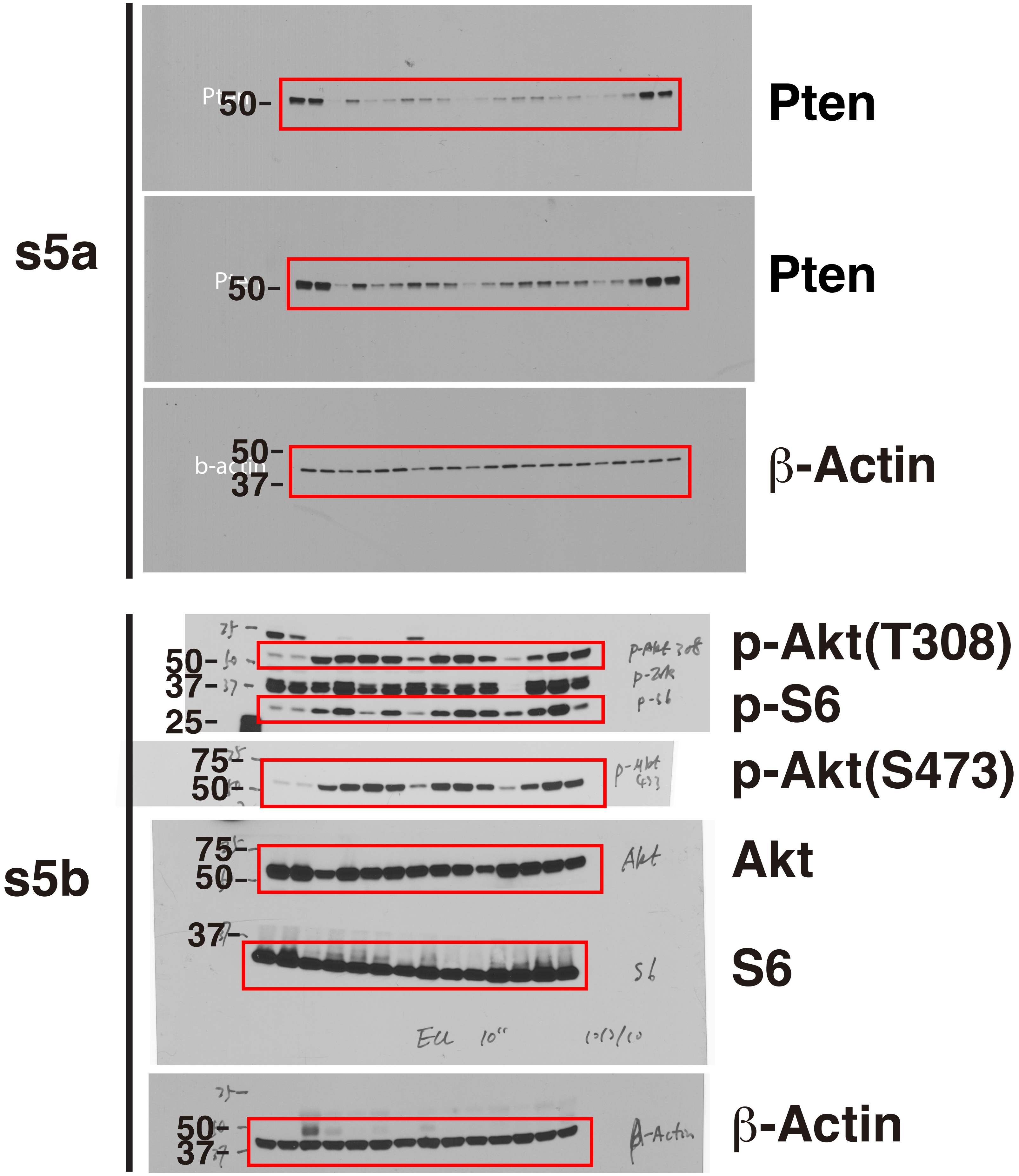

Uncropped immunoblots from Supplementary Figure 5

Supplementary Figure 7

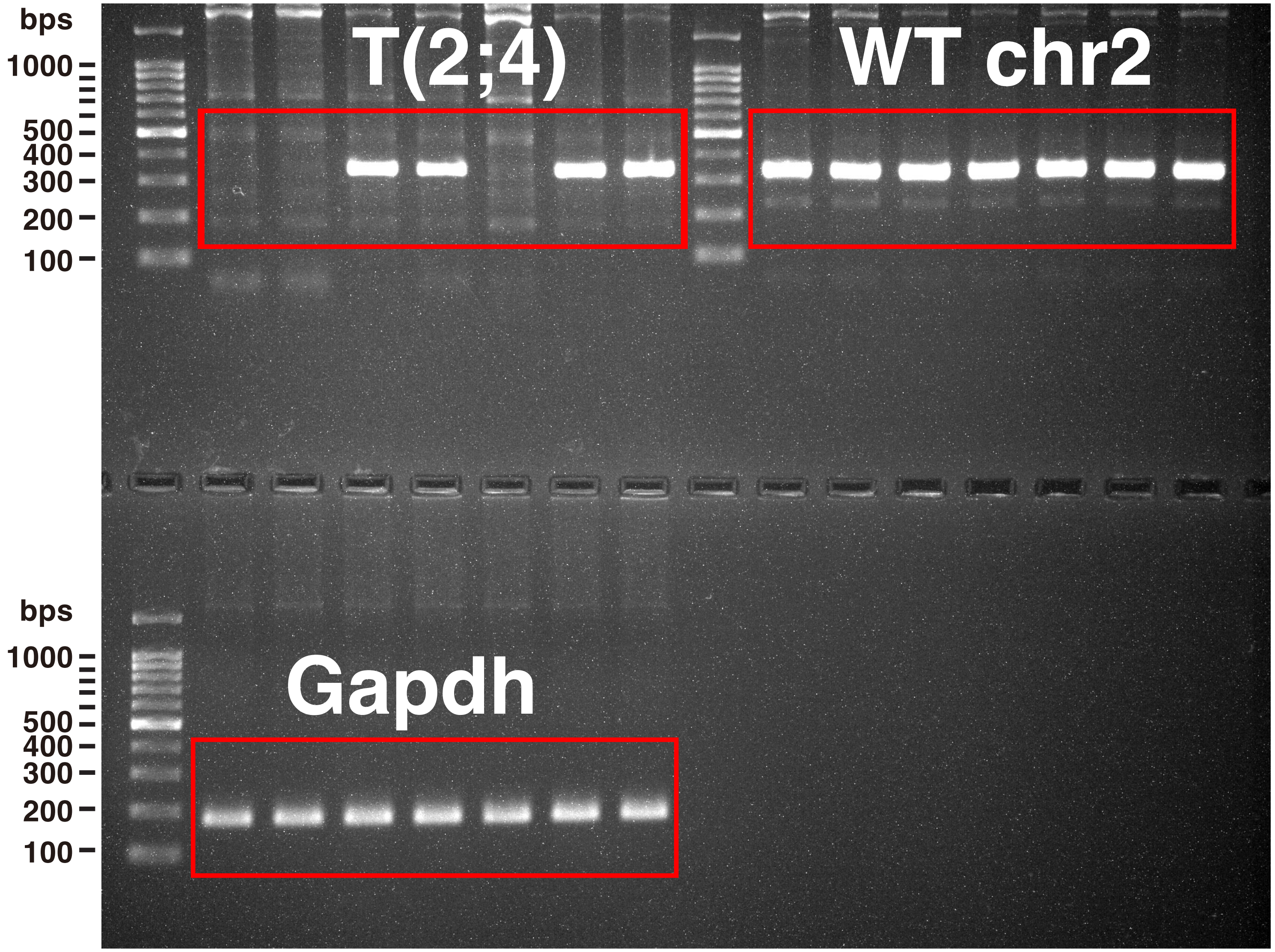

Uncropped DNA gel results from Supplementary Figure 7

Shared and private events in cancer drivers from WGS data in Mouse 2

Mouse 2 (Type 2)

Shared Events		Private Events			
SVZ[R] Tumor; SVZ[L] Tumor; Tumor 1 and Tumor 2	Tumor 1 and Tumor 2	SVZ[R] Tumor	SVZ[L] Tumor	Tumor 1	Tumor 2
chr11 qb5-qe1 loss (Nf1 del.)*	chr7 qA1-A2 loss*	Pik3cg gain*	Ptgfr loss* (chr3)	chr5 qG2-G3 gain*	Egfr-focal gain* (chr11)
chr4 qA1-qC4 loss (including Cdkn2a del.)*	chr11 qB4-E1 loss* (Trp53 loss)	chr17 qE4-E5 gain*	Sox1 gain* (chr8)	chr14 qA-B loss*	
t(2;4)*	Sox4/Cdkal1 gain* (chr13)		chr8 qA1.2 loss*		
Macrod2 loss* (chr2)			Map2k2 gain* (chr10)		
chr11 qB3 gain*			E2f7/Ifng loss* (chr10)		
chr11 qE2 focal loss*			chr11 qA1-A3 gain (Egfr)*		
			Tgif1 gain* (chr17)		

Original data for Figure 6

Shared and private events in cancer drivers from WGS data in Mouse 3

SVZ^{R} Tumor; Tumor R	SVZ^{R} Tumor	Tumor R
5 chr11 gains* (Mapk7, Ncor1, pik3r5/6)	chr11 qA4-aB1 gain*	Map2k2 gain* (chr10)
Nf1 focal deletion* (chr11)	chr14 qA2-qC2 gain*	Klf11 gain* (chr12)
Amp. Near Kif2b*		
Der(X)*		
Pik3ca^{H1047R}		

Original data for Figure 6

Shared and private events in cancer drivers from WGS data in Mouse 6

SVZ^{L}; Tumor and T_Early	T and T_Early	SVZ^{R}	SVZ^{L}	Tumor
13 gain or loss in chr11 (Ncor1 gain, Map2k4 loss, Trp53/Nf1, loss)*	chr12 qA1.1-qC1 loss*	Myc amp* (chr15)	3 loss in chr13*	chr15 qB3-qE2 deep gain* (Pdgfb, Ep300)
Gain and loss on part of chr14*	chr19 del between Sorcs1 and Ins1*	Lsamp promoter del*	Lsamp promoter del*	
Pten del*			4 events in chr16 (olig2 amp.)*	
chr19 end deletion*				
Nf1 splice site mutation				

Original data for Figure 6

Shared and private events in cancer drivers from WGS data in Mouse 5

	S5_3_SVZLP3	S5_4_SVZRP1_Tumor	S5_2_Primary Tumor/S5_5_TP1
	5 amp in chr15, including Myc*	Hras amp. (chr7)*	chr5, 7,10 gain, 19 loss
		End of chr7 amp. (chr7)*	21 events in chr7, including Hras/Ccnd1 amplification*
		Myo7a amp. (chr7)*	

Original data for Figure 6

Shared and private events in cancer drivers from WGS data in Mouse 10

S10_2_Primary Tumor 1; S10_6_T1P2	S10_3_Primary Tumor 2; S10_7_T2P4	S10_5_SVZP4	S10_6_T1P2	S10_7_T2P4	S10_2_Primary Tumor 1	S10_3_Primary Tumor 2
chr1 Cntnap5a intron loss*	Lrp1b intron loss*	chr8, 12, 15 loss	chr9 1st part gain*	chr2, 12 loss		chr10 end gain (Cdk4)*
chr2 Lrp1b intron loss*	chr8 Csmd1 intron loss*	Lrp1b loss*	2 chr11 events (Trp53)*	chr1 dqD-qE4 loss*		
5 chr7 qA1-qD1 gain* (Fgfr2)	2 chr7 loss *	8 chr3 gain/loss* (pik3ca, foxo1 gain)		chr4 2nd half loss *		
chr9 qA1-A3 loss*		1 chr4 loss*		Magi2 intron loss*		
		Magi2 loss*		chr10 2nd half gain (Cdk4)*		
		Dpp6 gain*		chr13 middle loss*		
		5 chr7 events*		3 events in chr16*		
		chr14 2nd half loss (Pbrm1, Rb1)*				
		2 chr16 events (Olig2 gain)*				

Original data for Figure 6

Shared and private events in cancer drivers from WGS data in Mouse 4

S4_2_SVZLP1; S4_3_SVZRP3; S4_4_T1P2; S4_5_T2P4	S4_3_SVZRP3; S4_4_T1P2; S4_5_T2P4	S4_2_SVZLP1	S4_3_SVZRP3	S4_4_T1P2	S4_5_T2P4
4 Chr6 short amplifications (Met, Ret, Ccnd2, Raf1)*	Macrod2 deletion(chr2)*	5 chr5 shallow deletions*	chr13 qD1-end amp (MAP3K1)*	chr2 qA1-qA3 gain (Notch1, Gata3)*	chr15 Stk32a gain*
Chr12 qA1-qB3 amplification (Ncoa1,Mycn)*	Grid2 promoter amp (chr6)*		chr11 qD-qE2 amp (Sox9)*	2 chr6 short deletions*	
	Ctnna2 deletion (chr6)*		chr15 qA1-qE2 amp*	2 chr15 amp (Myc)*	
	chr13 qA1-qA3.2 amplification (Hist1h4h, Irf4)*		chr19 qA amp*		
	Pbx1 L353H mut				

Original data for Figure 6
